# A comparison of methods to elicit causal structure

**DOI:** 10.3389/fcogn.2025.1544387

**Published:** 2025-05-21

**Authors:** Semir Tatlidil, Steven A. Sloman, Semanti Basu, Tiffany Tran, Serena Saxena, Moon Hwan Kim, Iris Bahar

**Affiliations:** ^1^Cognitive and Psychological Sciences, Brown University, Providence, RI, United States; ^2^Computer Science, Brown University, Providence, RI, United States; ^3^Computer Science, University of Mines, Boulder, CO, United States

**Keywords:** causality, causal Bayes nets, graphical models, counterfactual reasoning, mental representations

## Abstract

We compare two methods to elicit graphs from people that represent the causal structure of common artifacts. One method asks participants to focus narrowly on local causal relations and is based on the “make-a-difference” view of causality, specifically on an interventional theory of causality and so we call it “Intervention.” It asks subjects to answer a series of counterfactual questions. The second method draws directly from the graphical aspect of Causal Bayesian Networks and allows people to consider causal structure at a more global level. It involves drawing causal graphs using an online interface called “Loopy.” This method does not depend on a definition of causal relatedness. We use signal detection theory to analyze the likelihoods of people generating correct and incorrect causal relations (hit rates and false alarm rates, respectively) using each method. The results show that the intervention method leads people to generate more accurate causal models.

## Introduction

To use an object, it is sometimes necessary to understand how it works. For instance, it is hard to use a coffee machine without some idea of the function of each part. If you don't have that causal understanding yourself, you might need to rely on someone else's. Similarly, troubleshooting a problem depends on causal understanding and, when we do not have the causal knowledge ourselves, accessing someone else's is the next best thing. More generally, eliciting causal understanding is an important form of learning. To understand how a causal system works, we elicit causal understanding from experts. To find out if a student understands a causal system, we elicit their understanding. To collaborate with others to solve a problem involving causes and effects, we share causal understanding.

This paper focuses on one aspect of causal understanding, the causal structure that relates causes and effects to one another. Causal structure helps to identify invariants that are integral to a causal system and is thus of great utility. Indeed, asking people to provide structured causal understanding of a legal case in the form of a graph makes them more likely to endorse simpler legal explanations containing fewer cause-effect relations (Liefgreen and Lagnado, 2023). In other words, they become more sensitive to the value of parsimony.

Eliciting causal understanding requires a format to represent the relevant causal beliefs. To share my understanding of how a coffee machine works, it would be useful to have a language to express it. Expressing it in a natural language like English is one option, but has the drawback that every expression of the same causal system is likely to be very different because natural language offers so many ways to express the same idea; it is just too powerful. One consequence of this expressive diversity is that natural language expressions of two causal systems may not capture the actual similarity of those systems. My description of how a pen works and how a pencil works may be so different that the listener would fail to realize how similar pens and pencils are. Finally, natural language has no built-in facility for ensuring completeness. That is, it provides no cue that I may have failed to mention a relevant cause or effect or the relation between them.

To overcome these problems, we adopt a stripped-down version of a popular representational system for expressing causal understanding, Causal Bayes Nets (CBNs; Spirtes et al., 2001; Pearl, 2000). CBNs have been proposed as a cognitive model by philosophers (Glymour, 2001), computer scientists (Pearl, 2000), and cognitive scientists (e.g., Dasgupta et al., 2019; Sloman, 2005; cf. Waldmann, 2017). CBNs are types of Bayesian Networks that represent causes and effects as nodes, causal relations as directed edges from nodes representing causes to nodes representing effects, and include a special intervention operator (called the DO operator by Pearl) that allows the value of a variable to be set by an agent external to the causal system under scrutiny. The main virtue of CBNs is their ability to learn causal structure and make probabilistically correct inferences about the effects of actual and counterfactual interventions.

Rather than importing the mathematics of CBNs wholesale, we elicit causal understanding using simplified CBNs that better reflect how people actually think about causal systems. It is known that non-expert human causal understanding in most domains is shallow and superficial (Rozenblit and Keil, 2002; Sloman and Fernbach, 2017). Therefore, we elicit sets of causal relations that are unparameterized. They represent only qualitative causal structure, whether a causal relation exists between each pair of variables, not its strength in any sense. When an effect is produced by multiple causes, we do not demand that our subjects report the functional relation expressing how the causes lead to the effect. Thus, we only ask people to report causal structure at the high level at which actions and properties need to be described and not detailed causal structure. We refer to these abstract and simplified CBNs as “causal models.”

What form should causal structure elicitation take? We consider two forms that differ in the granularity that they demand people think about the causal system. One method is drawn directly from a popular class of theories of what comprises a causal relation and it asks participants to focus narrowly on local causal relations. The second method draws directly from the graphical property of CBNs and allows people to consider causal structure at a more global level.

Our first method relies on the “make-a-difference” view of causality (Lewis, 1973; Walsh and Sloman, 2011; Wolff, 2007), more specifically the interventional view championed by Woodward (2003). Woodward proposes that a causal relation is one that supports intervention. Roughly, A causes B if and only if an intervention by an external agent that sets A to some value results in B taking an associated value. The central idea is that, for A to be a cause of B, it must be more than correlated with B. The relation must be such that changing A by a sufficient amount also changes B. In that sense, A makes a difference to B. We will refer to this method as “Intervention.”

**Figure 1:**
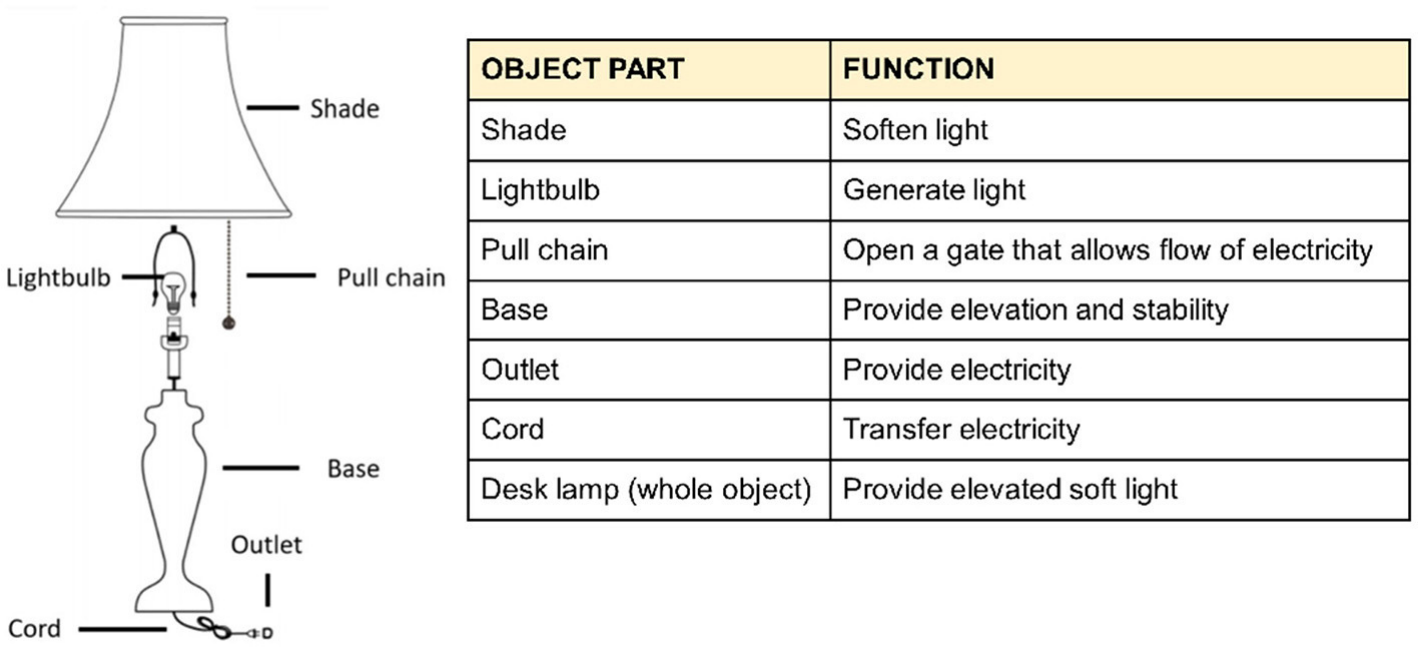
Diagram of desk lamp and a table describing the functions of its parts used in Experiment 1.

**Figure 2:**
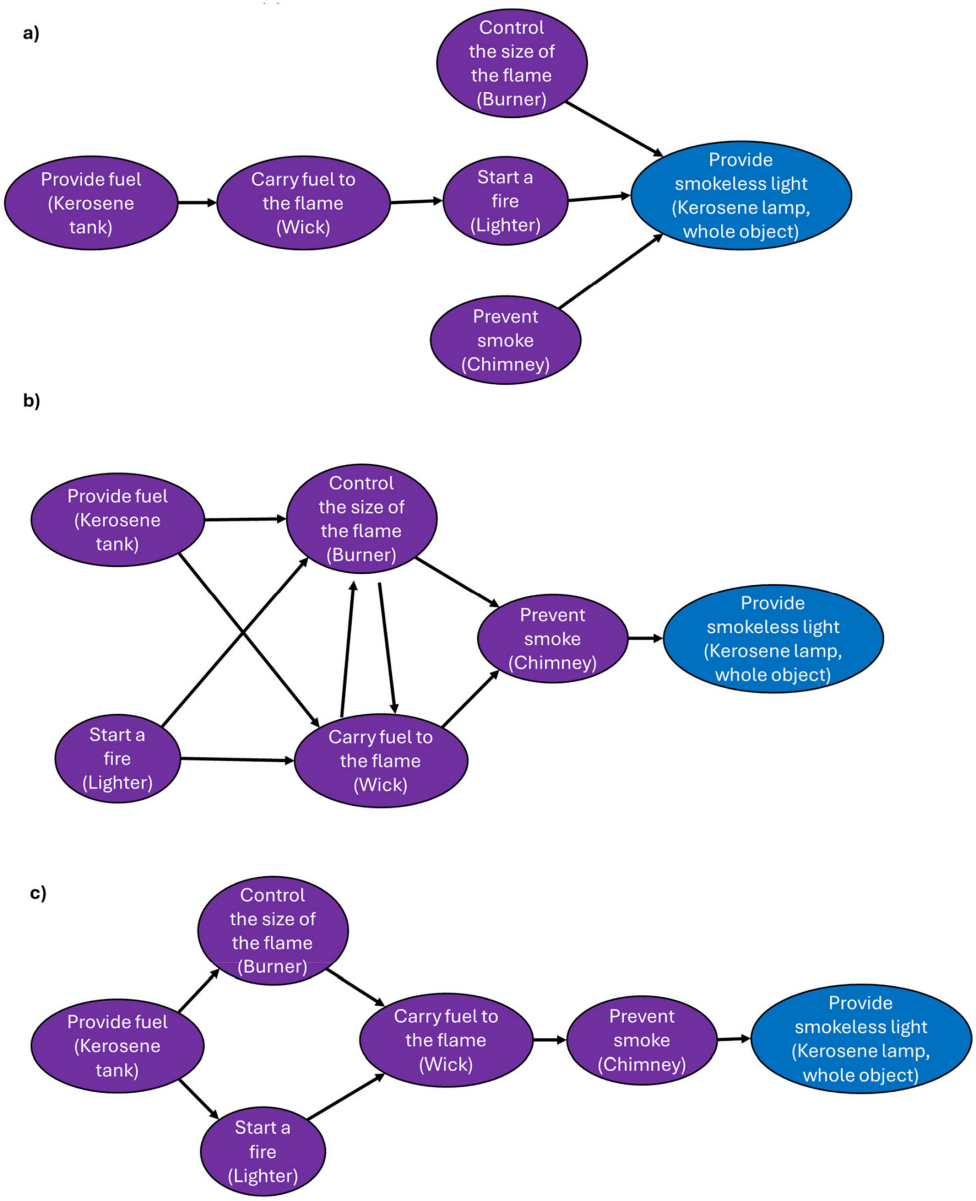
Example causal models of Kerosene Lamp from Experiment 1. **(a)** Causal model of a participant in the Drawing condition with *d*' = −0.16. **(b)** Causal model of a participant in the Intervention condition with *d*' = 1.77. **(c)** Ground truth causal model.

To implement it, we first break our causal system down into a set of component variables, each corresponding to a part of the causal system (see Figure 1 for an example). First we ask participants to imagine that the system is functioning normally. Then, for each pair of variables, we ask whether removing the first variable of the pair would result in the second variable still functioning. We assume that removing a variable corresponds to its associated part no longer functioning. We test the methods using a set of common objects like lamps, sinks, and bicycles. To illustrate the Intervention method, to elicit the causal structure of a desk lamp, one question we ask is “imagine that the outlet is removed, would the lightbulb still generate light?” If the subject answers “yes,” then we infer there is no causal relation between the two elements; a “no” implies that there is. For *n* elements, this produces a matrix of *n* – 1 × *n* – 1 responses (we assume variables are not causally related to themselves). In addition, we introduce an element that corresponds to the function of the whole object and serves only as an effect, never as a cause. From that matrix, we infer a causal structure from the set of causal relations on the assumption that two variables that are connected indirectly cannot also be connected directly. So if we obtain a causal chain from A to B to *C* then we assume there is no direct relation from A to C. We make this assumption because none of the parts in our objects serve as a both direct and an indirect cause of an effect. In cases where the link between A and B are bidirectional, we do not remove the direct link from A to *C* or B to C, as this would require us to arbitrarily choose whether we should treat A or B as the direct cause of C. An example causal model from a participant where we see such a structure is shown in Figure 2b, where there is both a direct link from wick to chimney and an indirect link through burner, but the direct link is not removed because the link between wick and burner is bidirectional.

This method has the virtue that it derives directly from a popular definition of causal relations in terms of counterfactuals (Gerstenberg et al., 2021) and requires that subjects consider only local, pairwise causality. It does have several drawbacks. First, it fails to measure the strength of a causal relation and any functional relations embedded in the causal structure. Second, it does not scale well. For an object with *N* parts, it requires *N*^2^ questions, a lot if *n* is large. Third, it cannot elicit the correct structure in the rare case that a variable is both a direct and an indirect cause of an effect.

Our second method (the “Drawing” condition) involves drawing causal graphs using an online interface called “Loopy”.^1^ Participants are given a graphical interface with all the elements of the causal system depicted as nodes. Their task is to draw arrows between nodes to represent causal structure. In essence, they are being asked directly to draw an unparameterized Bayesian Network although they are not restricted to acyclic graphs.

This method is less dependent on a definition of causal relatedness. Subjects may assume a counterfactual or interventional make-a-difference view or they may assume a more mechanistic understanding of causality. An example of the latter is Dowe's (1999) proposal that A causes B if and only if a change to A leads to a conserved quantity traveling from A to B over time and space with a resultant change in B. Lombrozo (2010) provides evidence that people take a mechanistic view in the case of physical causality but a make-a-difference view when thinking about intentional causality. Our graphical method also has the virtue that it allows people to think either locally about pairwise relations or more globally about several variables at a time. Finally, it scales better than the Intervention method because participants are free to consider how each variable influences all the others simultaneously. However, like the Intervention method, it does not pick up on all the properties of a CBN.

The purpose of the studies we report is to compare the efficacy of these two methods for eliciting causal structure. We evaluate the efficacy of the methods by comparing the causal models we elicit from participants to a “ground truth” causal model that is supposed to represent most closely how the object really works. We look at whether either method leads people to generate causal models that are closer to the ground truth models, and if either method introduces biases into the process of causal model elicitation.

## Experiment 1

### Methods

#### Participants

Two hundred and twenty six participants were recruited from Prolific. Thirty-one participants in the Drawing condition submitted causal graphs for all objects that either contained no links between nodes or used the function of the whole object as a cause which they were instructed not to do. These participants were excluded. Median age of the final sample was 37. Ninety identified as male, 100 as female, and 5 as other.

#### Materials and procedure

We elicited causal structure for four light-producing objects: a desk lamp, a wall lamp, a flashlight, and a kerosene lamp. For each object, participants saw a diagram of the object with its parts labeled alongside a table describing the function of each part (see Figure 1 for an example). The table also specified the function of the entire object. We included a function for the entire object in order to distinguish between parts that may be perceived as causally irrelevant from parts that may be perceived as causally relevant without having any direct links to other parts. As an example, for the desk lamp the function of the “base” is “provide elevation and stability” which is not causally linked to the functions of any other parts. We defined the function of the whole object as “provide elevated soft light” so that the base can be considered a cause of this function.

We included only object parts that were present in the vast majority of objects with that name. We only included parts that were most important for the object to function and parts that people were more likely to interact with. For instance, for the desk lamp, we omitted the socket because most desk lamps come with sockets pre-installed. The number of parts varied across objects from 4 to 6.

All participants began the experiment by completing a CAPTCHA to screen for bots. They were then asked to sign a consent form.

##### Intervention condition

Participants were first asked to commit to giving thoughtful answers (Geisen, 2022).^2^ Then they reviewed instructions explaining that the experiment was going to test their understanding of how objects work. They were told that they needed to answer a series of questions in the following format:

“If one removed the [X part] from the [object], would the [Y part] still [perform Y's function]?”

We used an example object (a pistol) that was not related to the test objects to explain how participants should answer the questions. For instance, we pointed out that if removing part X indirectly caused part Y to stop functioning by preventing an intermediate part from functioning, they should answer this question by saying “No”. Following the instructions, they saw an attention check question that asked what object was used as an example in the instructions. Then they proceeded to the experiment.

For each object, participants first saw a diagram of the object and a table describing the functions of its parts (see Figure 1). Then they were presented with the intervention questions for all permutations of pairs of object parts. We asked about the function of the whole object only as a potential effect of intervening on another part, but not as a potential cause of another part fulfilling its function. For example, we asked “If one removed the cord from desk lamp, would the desk lamp still provide elevated soft light”, but we never asked, “If one removed the desk lamp, would the cord still transfer electricity”. We ordered the questions in blocks corresponding to a specific part being removed. For instance, all questions involving “remove lightbulb” were asked after one another. The order of questions within these blocks, the order of blocks, and the order of objects were all counterbalanced across participants. The diagram, the table, and the questions were all on the same page, so participants were free to revisit the information about the object at any time.

##### Drawing condition

Participants were told that their task was to create causal models that explain how objects work in terms of the functional roles of the object parts. Using a spray bottle as an example, we gave a brief explanation of how graphs should be interpreted. For instance, they were told that each node represents a function of an object part, and an arrow from “X” to “Y” implies that “X” is a direct cause of “Y”. They were instructed to include all the relevant cause-effect relations for the whole object to fulfill its function in their graphs. They were also told that the node corresponding to the function of the whole object should only be used as an effect and not a cause. This restriction parallels the procedure in the intervention condition where participants were only asked about the function of the whole object as a potential effect. After this, they saw a demonstration of how the graphical interface worked before proceeding to the study.

Similar to the Intervention condition, participants in the Drawing condition first saw a diagram of the object and a table describing the functions of its parts. Then, they were asked to click a link to open the graphical interface on a separate page. By default, the interface displayed labeled nodes corresponding to the functions of each object part (and the whole object). Participants then had to draw links between nodes to create a causal model. They were also allowed to remove links they created and to restructure the graph visually, but they were prevented from making any other modifications such as removing or relabeling nodes. After creating the graph, they had to click a button on the interface to receive a link to their graph, which they had to paste into the Qualtrics survey page before proceeding to the next object. The order of objects was counterbalanced across participants. After completing all objects, participants in both conditions answered demographic questions.

### Results

Causal models from participants were excluded if they contained no causal links or contained a link that used the function of the whole object as a cause, as these violated our instructions. As will become clear, this biases against our conclusions.

To evaluate participants' causal models, we compare them to “ground truth” causal models. The structure of the ground truth models was agreed on by all authors and checked against expert knowledge found on the internet. To evaluate participants' models, we calculated hit rates (HR) and false alarm rates (FAR). They were defined as the following:


$$\begin{array}{l} HR\;=\\ \frac{\#\;of\;links\;present\;in\;both\;the\;ground\;truth\;and\;participant^{\prime }s\;model}{\#\;of\;links\;in\;the\;ground\;truth\;model} \end{array}$$

$$\begin{array}{l} FAR\;=\\ \frac{\#\;of\;links\;absent\;in\;the\;ground\;truth\;model\;that\;are\;present\;in\;the\;participant^{\prime }s\;model}{\#\;of\;links\;absent\;in\;the\;ground\;truth\;model} \end{array}$$

As shown in Table 1, the results were very clear. For all 4 objects, HRs were higher in the Intervention than the Drawing condition. FARs were also higher in the Intervention condition for 3 out of 4 objects, although one FAR difference (for flashlight) was small. Table 1 also lists the results of independent samples *t*-tests that compare HRs of each object across the two conditions and FARs of each object across the two conditions. The difference was highly significant in every case for HRs (*p* < 0.001) and in two of four cases for FARs. The average number of causal links was 4.69 in the Drawing condition, and 6.96 in the Intervention condition.

**Table 1:** Means with standard errors in parentheses for hit rates and false alarm rates across subjects in Experiment 1 for each object in both Drawing and Intervention conditions.

**Object**	**Drawing**		**Intervention**		***t*** **-tests**	
	**HR**	**FAR**	**HR**	**FAR**	**HR**	**FAR**
Desk lamp	0.61 (0.02)	0.08 (0.01)	0.78 (0.02)	0.14 (0.01)	*t*_(185)_ = −5.60^***^	*t*_(135.24)_ = −5.23^***^
Flashlight	0.71 (0.03)	0.1 (0.01)	0.91 (0.02)	0.12 (0.02)	*t*_(150.14)_ = −6.13^***^	*t*_(150.71)_ = −1.40
Kerosene lamp	0.29 (0.02)	0.17 (0.01)	0.61 (0.02)	0.3 (0.01)	*t*_(180.71)_ = −10.48^***^	*t*_(134.86)_ = −8.07^***^
Wall lamp	0.54 (0.02)	0.15 (0.01)	0.73 (0.02)	0.14 (0.01)	*t*_(187)_ = −6.75^***^	*t*_(152.86)_ = 0.74

*t*-tests comparing the two HRs and the two FARs in each row are also shown.

*t*-statistic not assuming homogeneity of variance was computed when Levene's test was significant. ^*^p < 0.05, ^**^p < 0.01, ^***^p < 0.001.

We conducted a 2 × 4 mixed ANOVA, with condition as a between-subjects factor and object as within-subjects factor. There was a significant effect of condition *F*_(1, 177)_ = 136.05, *p* < 0.001, $\eta_{p}^{2}$ = 0.44, where HR was lower in the Drawing condition (*M* = 0.54, SE = 0.01) than the Intervention condition (*M* = 0.75, SE = 0.01). There was also a significant effect of object *F*_(3, 531)_ = 115.32, *p* < 0.001, $\eta_{p}^{2}$ = 0.39, and a significant condition^*^object interaction effect *F*_(3, 531)_ = 5.87, *p* < 0.001, $\eta_{p}^{2}$ = 0.03.

We repeated the same analysis for FAR. There was a significant effect of condition *F*_(1, 177)_ = 21.52, *p* < 0.001, $\eta_{p}^{2}$ = 0.11, where FAR was lower in the Drawing condition (*M* = 0.12, SE = 0.00) than the Intervention condition (*M* = 0.18, SE = 0.01). Due to the object variable violating the assumption of sphericity (*p* < 0.001), F values were derived using the Huynh–Feldt statistic for the following: There was a significant effect of object *F*_(2.62, 464.01)_ = 62.53, *p* < 0.001, $\eta_{p}^{2}$ = 0.26, and a significant condition^*^object interaction *F*_(2.62, 464.01)_ = 15.65, *p* < 0.001, $\eta_{p}^{2}$ = 0.08.

The HRs and FARs indicate that the causal models that were elicited varied in their quality. Models of a flashlight elicited by the Intervention method were excellent while models of kerosene lamps were poor, especially when obtained with the Drawing method (see Figure 2 for examples).

Do these differences mean that the Intervention method provides a more sensitive method of eliciting causal relations than the Drawing method or do they mean that people are biased to report more causal relations with the Drawing method? To answer this question, we deployed signal detection theory (SDT; Green and Swets, 1966). SDT is a method developed in psychophysics to distinguish people's ability to detect a signal (e.g., a flash of light or evidence in memory for a causal relation) from any biases they may have to say “yes” or “no” when asked about the presence or absence of a signal. The ability to discriminate the presence of a signal from the absence of one for a given object in a given task is indicated by *d*', *z*(HR) – *z*(FAR). *d*' measures the similarity between a participant's causal model and the ground truth causal model. The more similar they are, the higher the *d*' is. An orthogonal measure, *c*, reflects any bias to report a causal link, averaged over when a causal link is present in the ground truth model and when it is absent: –[*z*(HR) + *z*(FAR)]/2. *c* = 0 means there is no bias in the task for that object, *c* < 0 means the subject is biased toward reporting a signal, *c* > 0 means a bias toward not reporting a signal.

One obstacle with using *d*' and *c* measures is that their values cannot be calculated when either the HR or FAR is either 1 or 0 because *z*(1) = ∞ and *z*(0) = –∞. We therefore replaced values of 1 with 0.99 and values of 0 with 0.01 when calculating *d*' and *c*.

Table 2 shows *d*' and *c* for each object using each elicitation method. The *d*' results in Table 2 make clear that, for every object, the Intervention condition led to better discrimination of true from imagined causal relations than the Drawing condition. The difference between the two *d*'s was significant in every case. They also show that subjects are more biased to report causal relations in the Intervention than the Drawing condition.

**Table 2:** Signal detection theory measures *d*' and *c* with standard errors in parentheses averaged across subjects in Experiment 1 for each object in both Drawing and Intervention conditions.

**Object**	**Drawing**		**Intervention**		***t*** **-tests**	
	***d*** **'**	**c**	***d*** **'**	**c**	***d*** **'**	**c**
Desk lamp	1.86 (0.11)	0.57 (0.03)	2.21 (0.1)	0.1 (0.05)	*t*_(177)_ = −2.28^*^	*t*_(151.26)_ = 8.53^***^
Flashlight	2.35 (0.19)	0.31 (0.03)	3.36 (0.14)	−0.16 (0.05)	*t*_(152.83)_ = −4.20^***^	*t*_(167.53)_ = 7.75^***^
Kerosene lamp	0.32 (0.09)	0.82 (0.02)	0.94 (0.08)	0.12 (0.06)	*t*_(177)_ = −5.18^***^	*t*_(127.78)_ = 10.96^***^
Wall lamp	1.15 (0.11)	0.51 (0.03)	2.15 (0.14)	0.23 (0.04)	*t*_(174.55)_ = −5.71^***^	*t*_(173.22)_ = 5.40^***^

*t*-tests comparing the two d's and the two c's in each row are also shown.

^*^ denotes *p* < 0.05.

^**^ denotes *p* < 0.01.

^***^ denotes *p* < 0.001.

To investigate these effects using all the data on *d*', we conducted a 2 × 4 mixed ANOVA, with condition as a between-subjects factor and object as a within-subjects factor. There was a significant effect of condition *F*_(1, 177)_ = 52.06, *p* < 0.001, $\eta_{p}^{2}$ = 0.23 where *d*' was lower in the Drawing condition (*M* = 1.46, SE = 0.08) than the Intervention condition (*M* = 2.14, SE = 0.07). Due to the object variable violating the assumption of sphericity (*p* < 0.001), F values were derived using the Huynh-Feldt statistic for the following: a significant effect of object *F*_(2.66, 471.17)_ = 122.37, *p* < 0.001, $\eta_{p}^{2}$ = 0.41 and a significant condition^*^object interaction *F*_(2.66, 471.17)_ = 3.72, *p* = 0.015, $\eta_{p}^{2}$ = 0.02. All ANOVAs met the assumption of sphericity throughout the rest of the paper, unless mentioned explicitly.

We repeated the same analysis for c. There was a significant effect of condition *F*_(1, 177)_ = 161.70, *p* < 0.001, $\eta_{p}^{2}$ = 0.48 where *c* was higher in the Drawing condition (*M* = 0.55, SE = 0.02) than the Intervention condition (*M* = 0.08, SE = 0.03). Due to the object variable violating the assumption of sphericity (*p* < 0.05), F values were derived using the Huynh–Feldt statistic for the following: a significant effect of object *F*_(2.94, 519.73)_ = 35.02, *p* < 0.001, $\eta_{p}^{2}$ = 0.17 and a significant condition^*^object interaction *F*_(2.94, 519.73)_ = 9.13, *p* < 0.001, $\eta_{p}^{2}$ = 0.05.

Experiment 1 shows clearly that the Intervention method leads to a greater bias to report causal links than the Drawing method. More importantly, it generates causal models that are more discriminating. The causal relations they elicit are relatively more likely to be true causal relations than incorrect ones.

## Experiment 2

Experiment 2 attempts to replicate the results of Experiment 1 using more and more complex objects.

### Methods

#### Participants

Two hundred and fifty five participants were recruited from Prolific. Twenty-two participants in the Drawing condition submitted causal graphs for all objects that either contained no links between nodes or used the function of the whole object as a cause, which they were instructed not to do. These participants were excluded. Median age of the final sample was 35. One hundred and twenty one identified as male, 108 as female, and 4 as non-binary.

#### Materials and procedure

The methods of Experiment 2 were identical to Experiment 1 except for the following. We elicited causal models for 10 objects: bicycle, cannon, electric mixer, hand mixer, paddle boat, pistol, scooter, sink, toilet, tricycle. The number of object parts ranged from 5 (hand mixer) to 12 (bicycle). We changed the tutorial object used in the Intervention question from pistol to desk lamp, as we used pistol as a test object in this experiment.

### Results

In this experiment, participants were exposed to only 3 out of 10 randomly chosen objects. This prevents us from running the same ANOVA analysis that we ran for Experiment 1 as it requires participants to undergo every level of the within-subjects factor. Instead, to analyze the effect of condition on HR, we calculated an average HR score for each participant by taking the mean HR of their 3 objects. Then we ran an independent samples *t*-test on average HR. This revealed a highly significant effect of condition *t*_(202.20)_ = −12.51, *p* < 0.001, *d* = −1.68, where HR was lower in the Drawing condition (*M* = 0.29, SE = 0.02) than the Intervention condition (*M* = 0.54, SE = 0.01). The same analysis on average FAR scores also showed a highly significant effect of condition *t*_(168.36)_ = −13.95, *p* < 0.001, *d* = −1.67. FAR was lower in the Drawing condition (*M* = 0.09, SE = 0.00) than the Intervention condition (*M* = 0.23, SE = 0.01). The average number of causal links was 8.40 in the Drawing condition, and 21.82 in the Intervention condition.

These results reveal the same pattern as Experiment 1. As indicated in Table 3, for every one of the 10 objects, both HRs and FARs were higher in the Intervention than the Drawing condition. Table 3 also lists the results of two *t*-tests for each object, one comparing HRs and the other comparing FARs in the two conditions. For HRs, the difference was significant (*p* < 0.001) in every case but one (for sink). For FARs, all differences between the conditions were significant.

**Table 3:** Means with standard errors in parentheses for hit rates and false alarm rates across subjects in Experiment 2 for each object in both Drawing and Intervention conditions.

**Object**	**Drawing**		**Intervention**		***t*** **-tests**	
	**HR**	**FAR**	**HR**	**FAR**	**HR**	**FAR**
Bicycle	0.25 (0.05)	0.07 (0.01)	0.56 (0.02)	0.26 (0.02)	*t*_(28.81)_ = −5.30^***^	*t*_(43.09)_ = −9.83^***^
Cannon	0.26 (0.02)	0.10 (0.0)	0.50 (0.02)	0.30 (0.03)	*t*_(61)_ = −7.07^***^	*t*_(38.43)_ = −7.38^***^
Electric mixer	0.26 (0.02)	0.09 (0.0)	0.45 (0.03)	0.26 (0.03)	*t*_(57)_ = −4.69^***^	*t*_(36.75)_ = −6.50^***^
Hand mixer	0.42 (0.03)	0.13 (0.01)	0.56 (0.03)	0.25 (0.02)	*t*_(72)_ = −2.88^**^	*t*_(51.85)_ = −4.98^***^
Paddle boat	0.32 (0.04)	0.09 (0.01)	0.60 (0.03)	0.16 (0.01)	*t*_(61)_ = −5.37^***^	*t*_(54.67)_ = −4.71^***^
Pistol	0.25 (0.03)	0.06 (0.01)	0.52 (0.02)	0.20 (0.02)	*t*_(70)_ = −7.48^***^	*t*_(44.61)_ = −6.12^***^
Scooter	0.15 (0.03)	0.12 (0.01)	0.52 (0.03)	0.17 (0.02)	*t*_(62.66)_ = −8.86^***^	*t*_(53.77)_ = −2.26^**^
Sink	0.57 (0.07)	0.06 (0.01)	0.68 (0.03)	0.22 (0.02)	*t*_(33.86)_ = −1.46	*t*_(56.42)_ = −7.40^***^
Toilet	0.24 (0.03)	0.07 (0.01)	0.50 (0.02)	0.23 (0.02)	*t*_(57)_ = −5.94^***^	*t*_(48.70)_ = −9.53^***^
Tricycle	0.18 (0.04)	0.12 (0.01)	0.47 (0.04)	0.26 (0.02)	*t*_(61)_ = −4.77^***^	*t*_(51.67)_ = −5.48^***^

*t*-tests comparing the two HRs and the two FARs in each row are also shown.

^*^ denotes *p* < 0.05.

^**^ denotes *p* < 0.01.

^***^ denotes *p* < 0.001.

Does the Intervention method provide a more sensitive method of eliciting causal relations than the Drawing method or does it induce people to report more causal relations? We again address this question with SDT. Using the average *d*' as our DV, we found a significant effect of condition *t*_(171.09)_ = −2.89, *p* = 0.004, *d* = −0.40, where *d*' was lower in the Drawing condition (*M* = 0.67, SE = 0.07) than the Intervention condition (*M* = 0.92, SE = 0.04). For the *c* measure, there was a significant effect of condition *t*_(213.89)_ = 17.31, *p* < 0.001, *d* = 2.29, where *c* was higher in the Drawing condition (*M* = 1.06, SE = 0.03) than the Intervention condition (*M* = 0.35, SE = 0.03). For the average HR, FAR, and *d*' measures, Levene's test was significant so the *t* statistics were computed without assuming equal variances.

*d*' and *c* measures for each object are reported in Table 4. Again, *d*' tends to be higher in the Intervention than Drawing condition, however the difference is only significant for 3 objects: paddle, scooter, and tricycle. For c, all differences are significant.

**Table 4:** *d*' and *c* with standard errors in parentheses averaged across subjects in Experiment 2 for each object in both Drawing and Intervention conditions.

**Object**	**Drawing**		**Intervention**		***t*** **-tests**	
	***d*** **'**	***c***	***d*** **'**	***c***	***d*** **'**	***c***
Bicycle	0.43 (0.26)	1.27 (0.1)	0.82 (0.08)	0.28 (0.05)	*t*_(24.34)_ = −1.43	*t*_(28.79)_ = 8.70^***^
Cannon	0.54 (0.14)	1.03 (0.06)	0.59 (0.1)	0.30 (0.05)	*t*_(61)_ = −0.31	*t*_(61)_ = 9.25^***^
Electric mixer	0.67 (0.1)	1.01 (0.04)	0.54 (0.1)	0.41 (0.07)	*t*_(57)_ = 0.86	*t*_(51.80)_ = 7.55^***^
Hand mixer	0.93 (0.18)	0.75 (0.04)	0.91 (0.1)	0.30 (0.07)	*t*_(57.46)_ = 0.08	*t*_(60.14)_ = 5.73^***^
Paddle boat	0.82 (0.21)	1.02 (0.07)	1.32 (0.11)	0.37 (0.05)	*t*_(39.16)_ = −2.15^*^	*t*_(61)_ = 7.70^***^
Pistol	0.80 (0.15)	1.20 (0.06)	0.96 (0.07)	0.44 (0.05)	*t*_(44.91)_ = −1.01	*t*_(70)_ = 9.60^***^
Scooter	0.01 (0.16)	1.20 (0.07)	1.15 (0.13)	0.51 (0.07)	*t*_(63)_ = −5.17^***^	*t*_(63)_ = 5.99^***^
Sink	2.02 (0.34)	0.69 (0.1)	1.38 (0.12)	0.14 (0.07)	*t*_(27.24)_ = 1.76	*t*_(63)_ = 4.55^***^
Toilet	0.70 (0.14)	1.14 (0.07)	0.79 (0.07)	0.39 (0.05)	*t*_(57)_ = −0.66	*t*_(57)_ = 8.29^***^
Tricycle	0.00 (0.22)	1.23 (0.09)	0.62 (0.12)	0.39 (0.08)	*t*_(61)_ = −2.73^**^	*t*_(61)_ = 6.50^***^

*t*-tests comparing the two d's and the two c's in each row are also shown.

^*^ denotes *p* < 0.05.

^**^ denotes *p* < 0.01.

^***^ denotes *p* < 0.001.

As an exploratory analysis, we looked at the correlation between the number of object parts and the HR, FAR, and *d*' measures to see if the variation between objects was related to the complexity of the object. We found that objects with more parts resulted in lower HR *r*_(639)_ = −0.10, *p* = 0.009. However, the number of object parts did not significantly correlate with FAR *r*_(639)_ = −0.03, *p* = 0.488, or *d*' *r*_(639)_ = −0.08, *p* = 0.059.

## General discussion

Of the two methods we compared for eliciting causal structure, the Intervention method elicited more accurate causal models than the Drawing method in the sense that Interventional models more effectively discriminated true from false causal links. The Intervention method was also associated with a higher bias to report causal links compared to the Drawing method.

In both cases, participants were given a set of parts along with associated functions and they were asked to generate causal links among the parts. The Drawing method made use of an open-source tool to allow participants to generate causal graphs in whatever way they preferred. They were free to consider each pair of parts or to think about multiple parts simultaneously. It also did not constrain their interpretation of causality. They were asked to draw all the cause-effect relations for the whole object that allowed it to fulfill its function. They could think about causal relations in terms of a mechanism (the transfer of some conserved quantity from causes to effects), a relation that supports intervention, or indeed any sort of dependency relation that supports the counterfactual, “The cause and effect both obtained and, if the cause had not been present, then the effect would not have occurred.”

The Intervention method asked a series of counterfactual questions, essentially one for each pair of parts, and a causal model was inferred for each object from each subject's answers. By virtue of asking this series of questions, this method presupposes that causal relations support counterfactuals and, in that sense, depends on a more constrained definition of causality than the Drawing method. The method also poses questions about pairs of variables and thus involves a more local perspective on causal relations; i.e., one pair of parts at a time.

Why did the Intervention method generate more and more discriminating causal relations? Is it because of its more local perspective compared to the Drawing method, to its more constrained interpretation of causality, or some other difference between the two procedures? Although our experiments were not designed to answer this question, we guess that it is the more local perspective of the Intervention method. Our reasoning is that the method forces people to consider each pair of parts and is thus more likely to induce them to consider causal links that they might otherwise have overlooked. Nothing in the Drawing method forces this kind of exhaustive consideration of the space of possible causal relations. Furthermore, we doubt that how people think about causal relations (as mechanisms or dependencies) is likely to have much effect on their reports. Data from other domains of cognition lead us to suspect that people are not even aware of how they are thinking about causal relatedness (Nisbett and Wilson, 1977).

The Intervention method is better at generating more complete and accurate causal models than the Drawing method, but this benefit does come at a cost. The Intervention method does not scale as well as the number of parts increases. If there are *N* parts, the Intervention method requires asking *N*^2^ questions and this can be laborious if *N* is large. For reasonable *N*, it is manageable because questions can be grouped as we grouped them for maximum efficiency. Each of the *N* causes can be considered (“If one removed [X part] from [object],…”) with a list of its *N* potential effects, (“would [Y part] still [perform Y's function]?”), so subjects only have to imagine removing the cause once, while ticking off all of the effects of that removal. Nevertheless, the Drawing method is less vulnerable to this scaling issue as it allows subjects to think about connecting parts in whatever way they want, including as clusters of causes and effects.

The notion of causality we are eliciting focuses on how an object actually operates rather than an object part's potential to operate. For instance, the ground truth model for the kerosene lamp (Figure 2c) indicates that in order for the chimney to prevent smoke, fuel and fire are necessary because without them, there would be no smoke for the chimney to prevent. However, this does not imply that the chimney loses its ability to prevent smoke. Our causal models are meant to capture the events involved in how the object functions, and the event of “prevent smoke” cannot occur in the absence of smoke.

In conclusion, causal structure determines how the world works and therefore how we make predictions, generate explanations, troubleshoot problems, and many of the myriad cognitive tasks that we perform. The ability to elicit causal structure is therefore fundamental to understanding how people think about the world. Our studies suggest that a method that relies on imagined interventions produces more accurate results than one that relies on drawing graphs. One theoretical implication of these findings is that people do not necessarily store fixed mental representations of causal systems. Instead, these representations are constructed in response to the method used to elicit them. The practical applications of these results are illustrated in our own work. We used causal models generated by participants to improve robot planning algorithms to aid in tasks such as object assembly and troubleshooting (Basu et al., 2025, 2024). More broadly, there are many real-life situations where people need to explicitly represent or use causal information to make decisions, such as jurors reaching a verdict (Liefgreen and Lagnado, 2023), decisions about one's own health (Kleinberg et al., 2023), or personal finances (Korshakova et al., 2023). Our results indicate the method of presenting causal information can impact people's causal beliefs, and in turn, their decisions.

## Data Availability

The raw data supporting the conclusions of this article will be made available by the authors, without undue reservation.

## Footnotes

^1^Loopy is an open-source online software (see: https://ncase.me/loopy/). We used a modified version of Loopy that prevented participants from making unwanted modifications to the graphs as explained in the Methods section.

^2^After the experiment, we worried that participants may interpret this question as a sign of researchers not trusting them, and thus we removed it from Drawing condition in Experiment 1 and both conditions in Experiment 2.
