# Supplementary material for: A comparison of methods to elicit causal structure
Source: Front Cognit. 2025 May 21;4:1544387. doi: 10.3389/fcogn.2025.1544387 (PMC13281103; doi:10.3389/fcogn.2025.1544387)
Supplement: Supplementary file 1 [file Table_1.docx]

**Supplementary Materials for “A Comparison of Methods to Elicit Causal Structure”**

1. The diagrams, part/function tables, and the ground truth causal models for all objects used in Experiment 1.
2. The diagrams, part/function tables, and the ground truth causal models for all objects used in Experiment 2.
3. **The diagrams, part/function tables, and the ground truth causal models for all objects used in Experiment 1.**

**Figure S1**


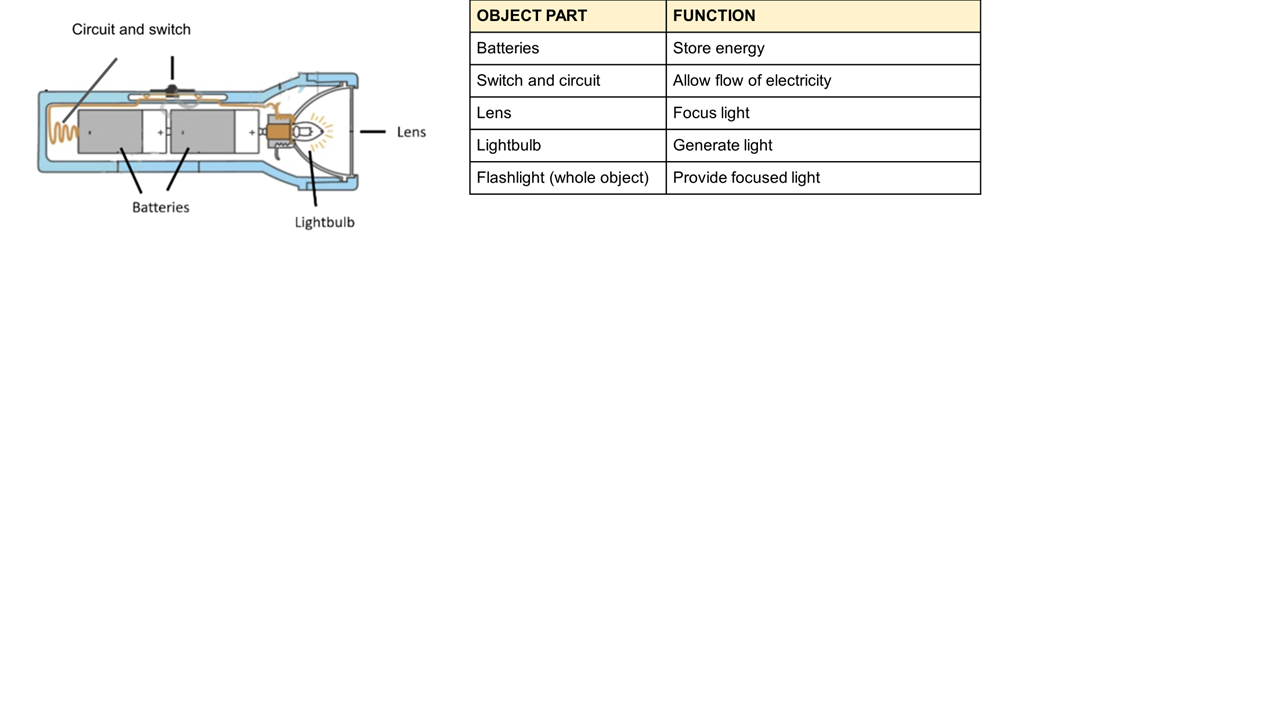

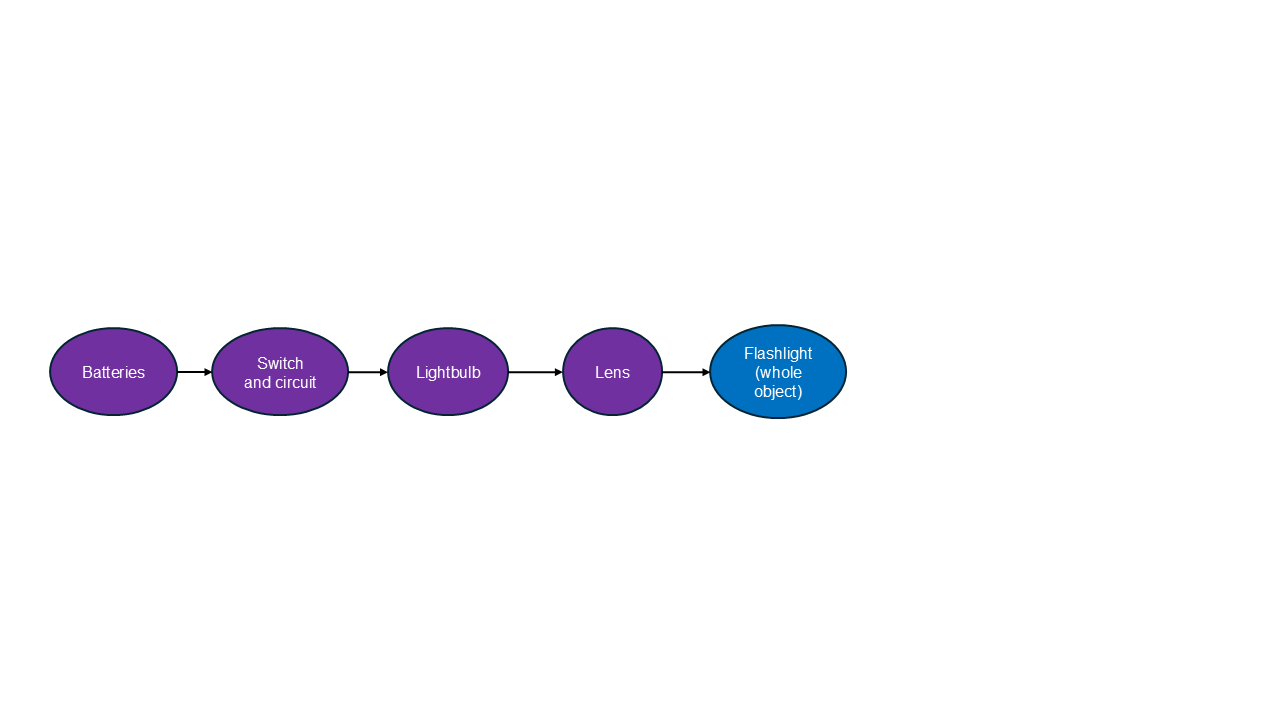
The diagram, part/function table, and the ground truth causal model for the flashlight.

**Figure S2**


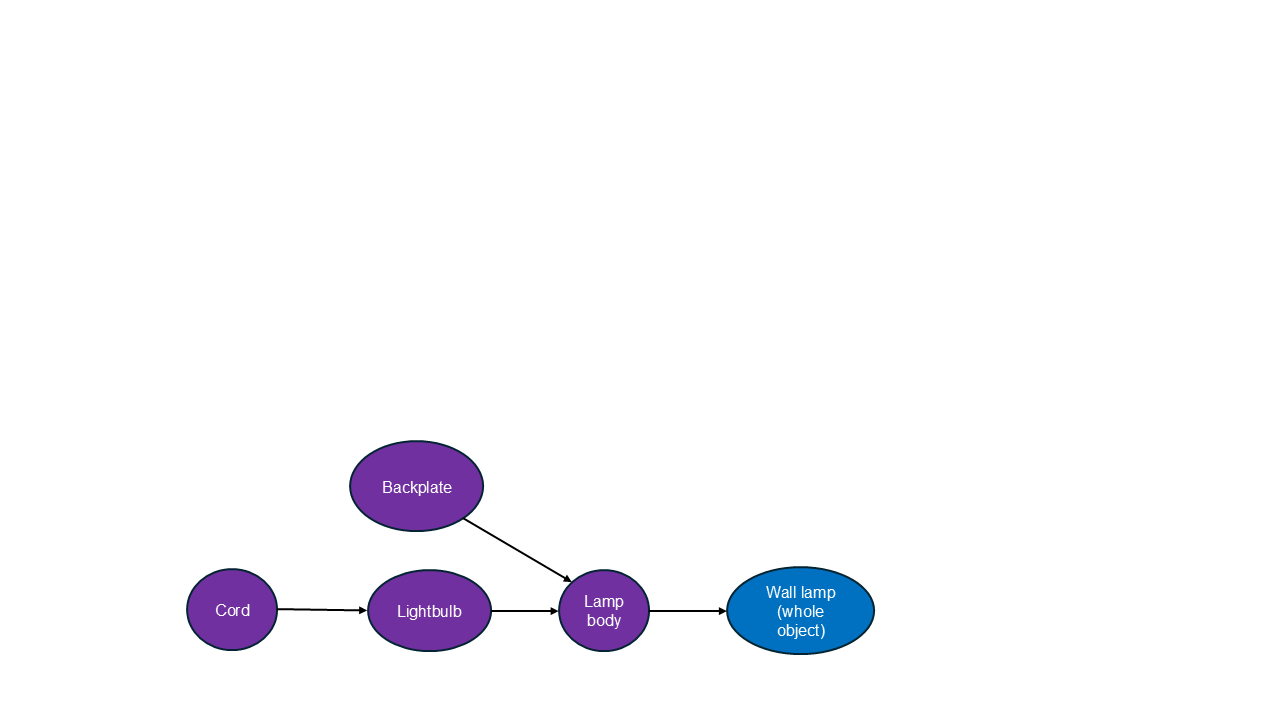

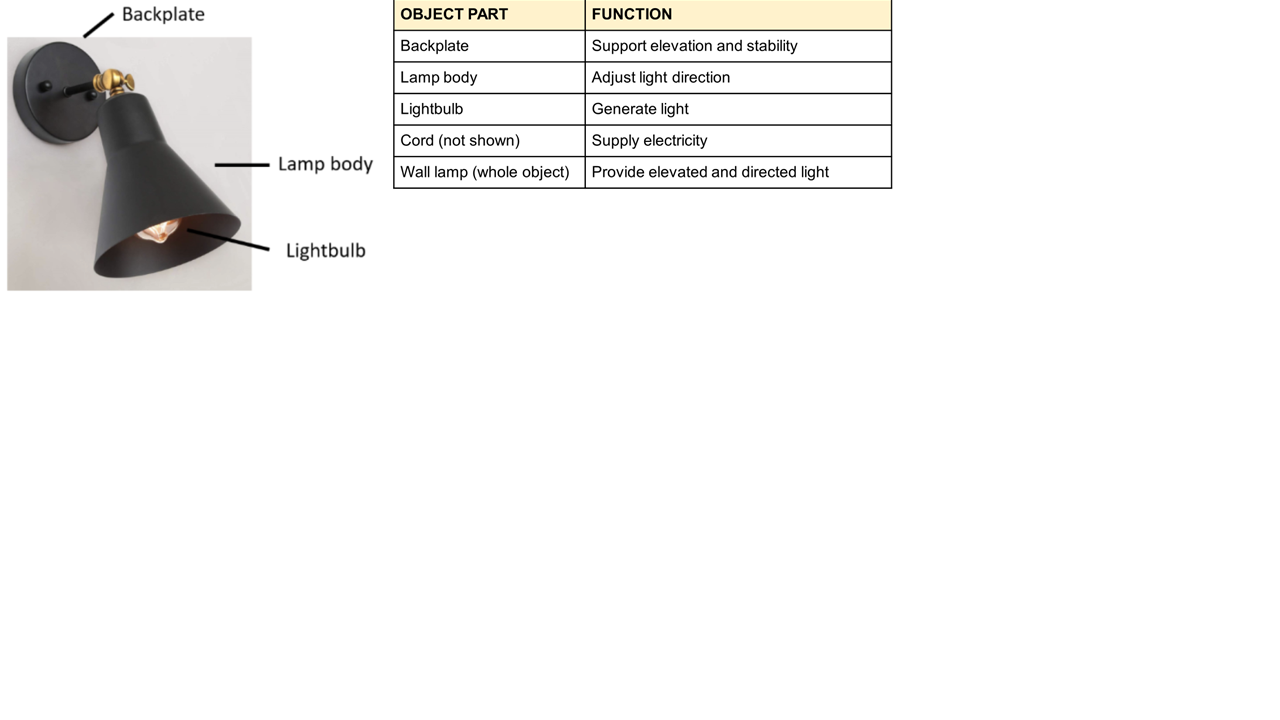
The diagram, part/function table, and the ground truth causal model for the wall lamp.

**Figure S3**


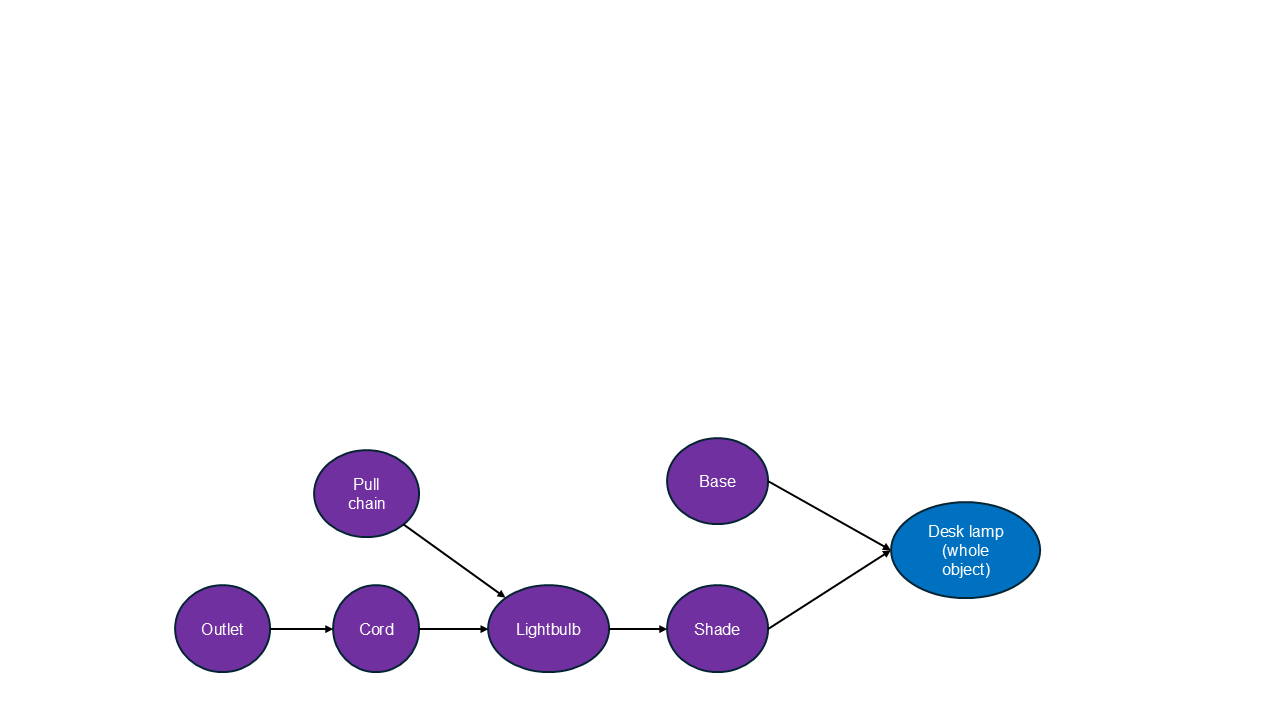

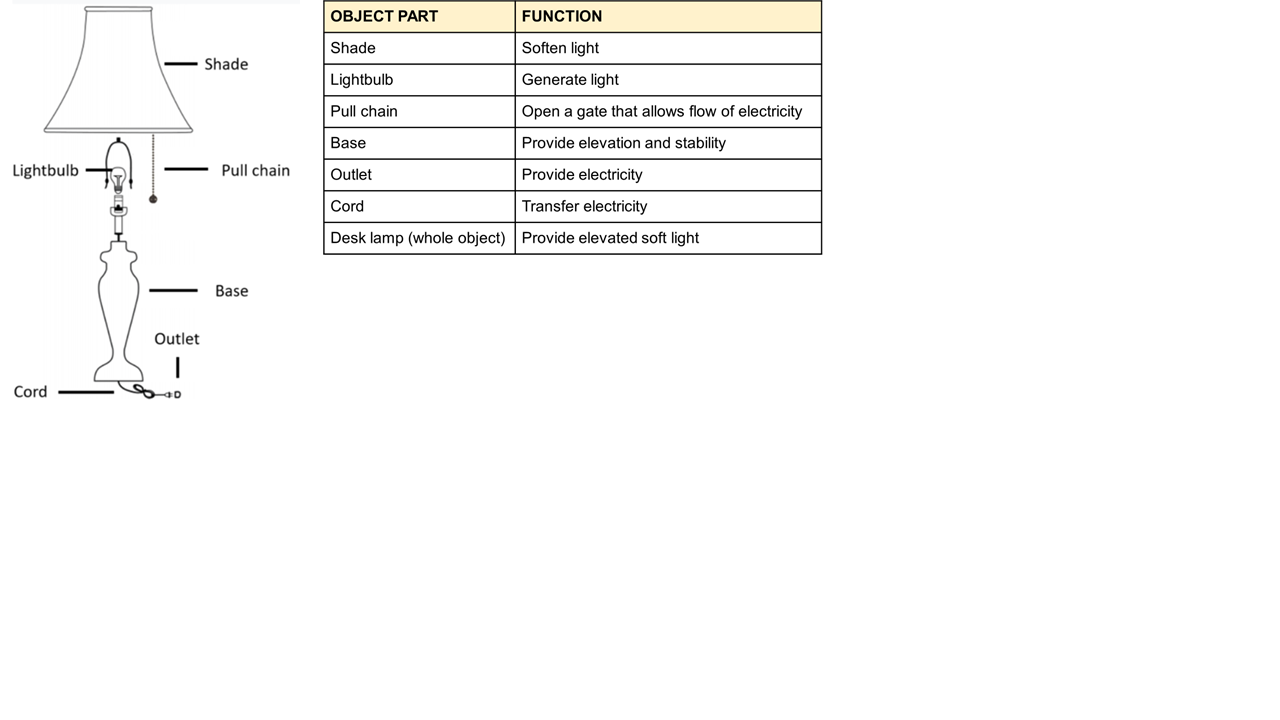
The diagram, part/function table, and the ground truth causal model for the desk lamp.

**Figure S4**


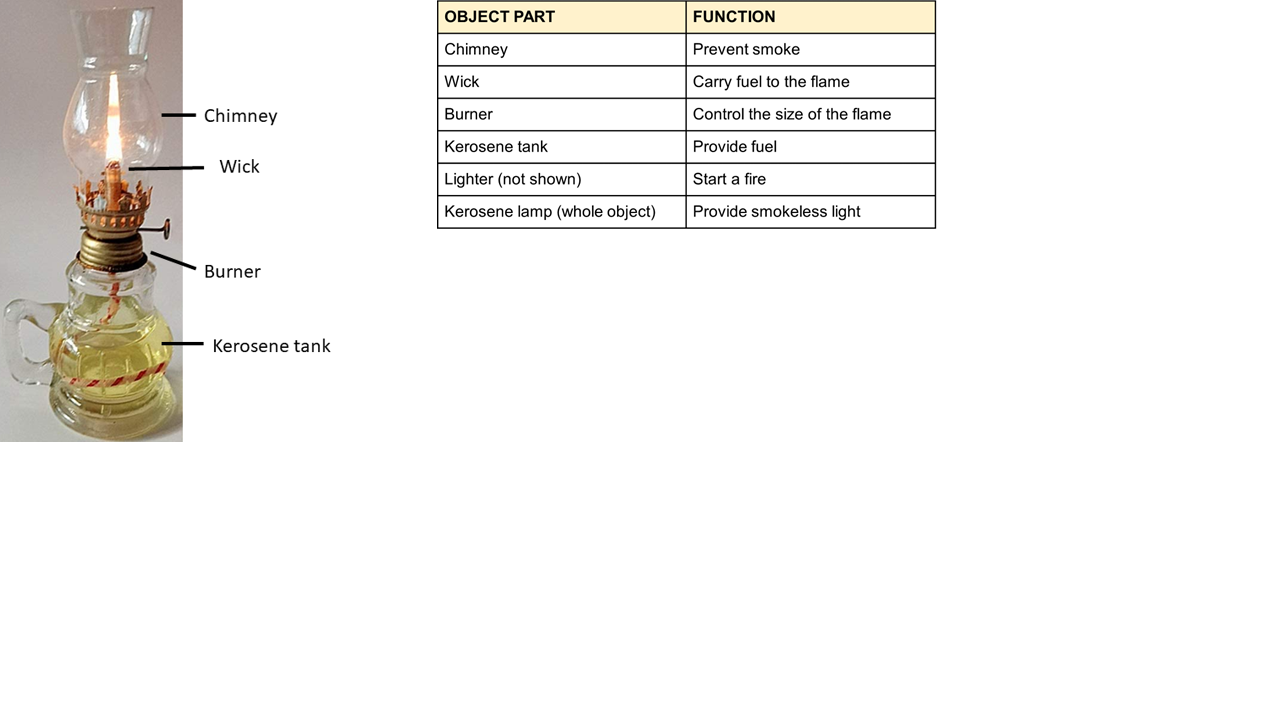
The diagram, part/function table, and the ground truth causal model for the kerosene lamp.
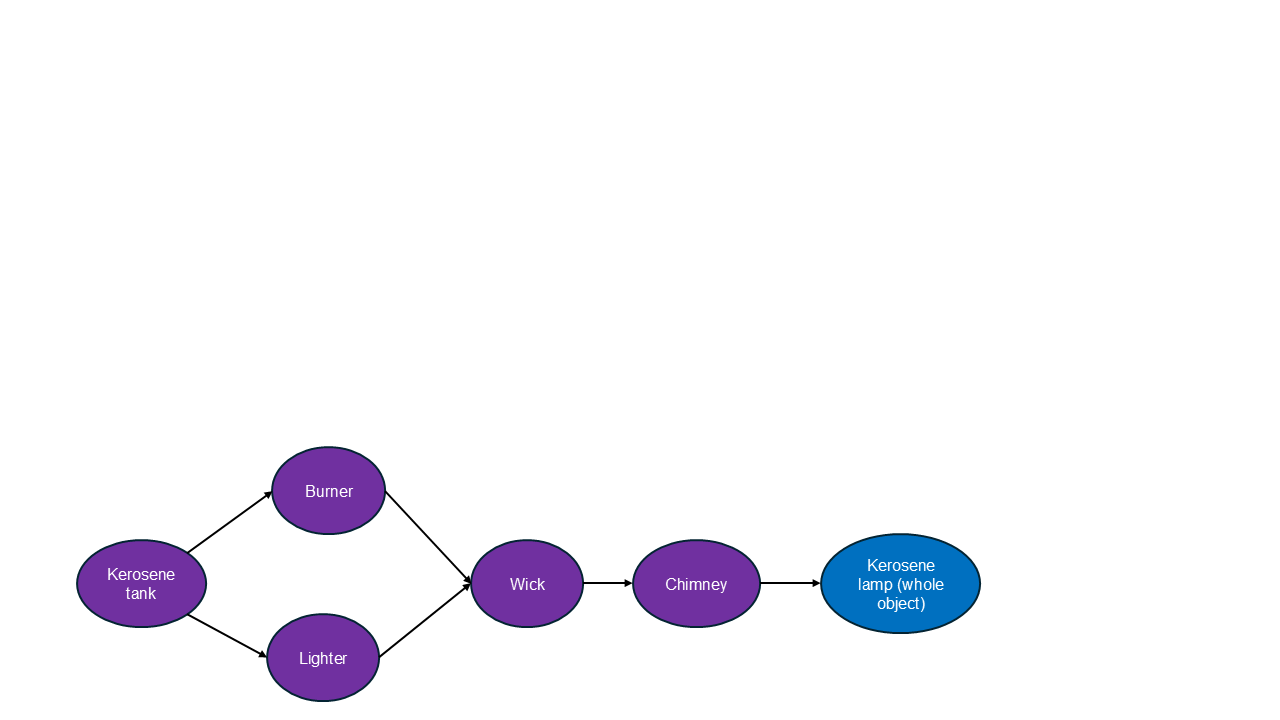


1. **The diagrams, part/function tables, and the ground truth causal models for all objects used in Experiment 1.**

**Figure S5**


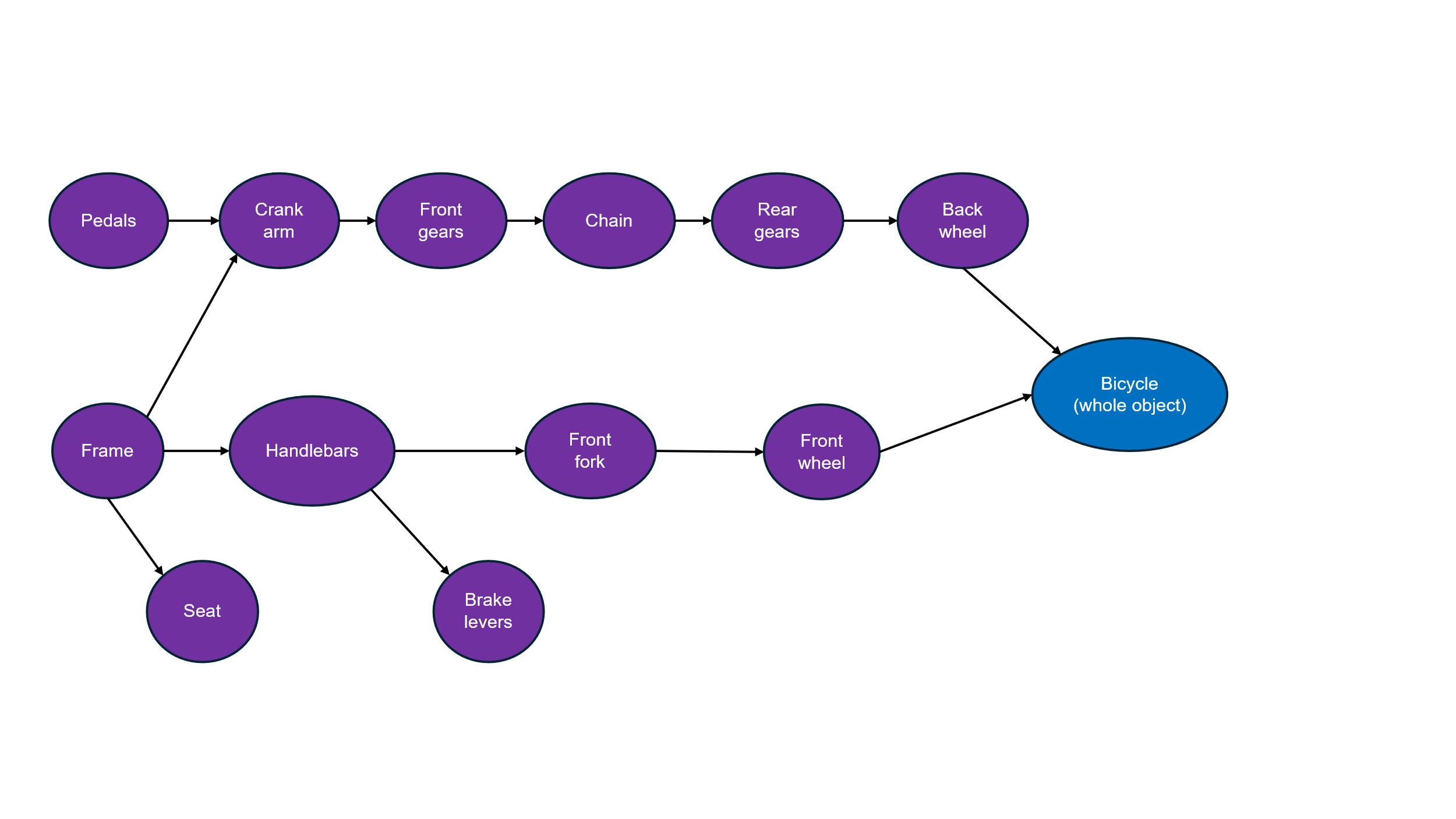

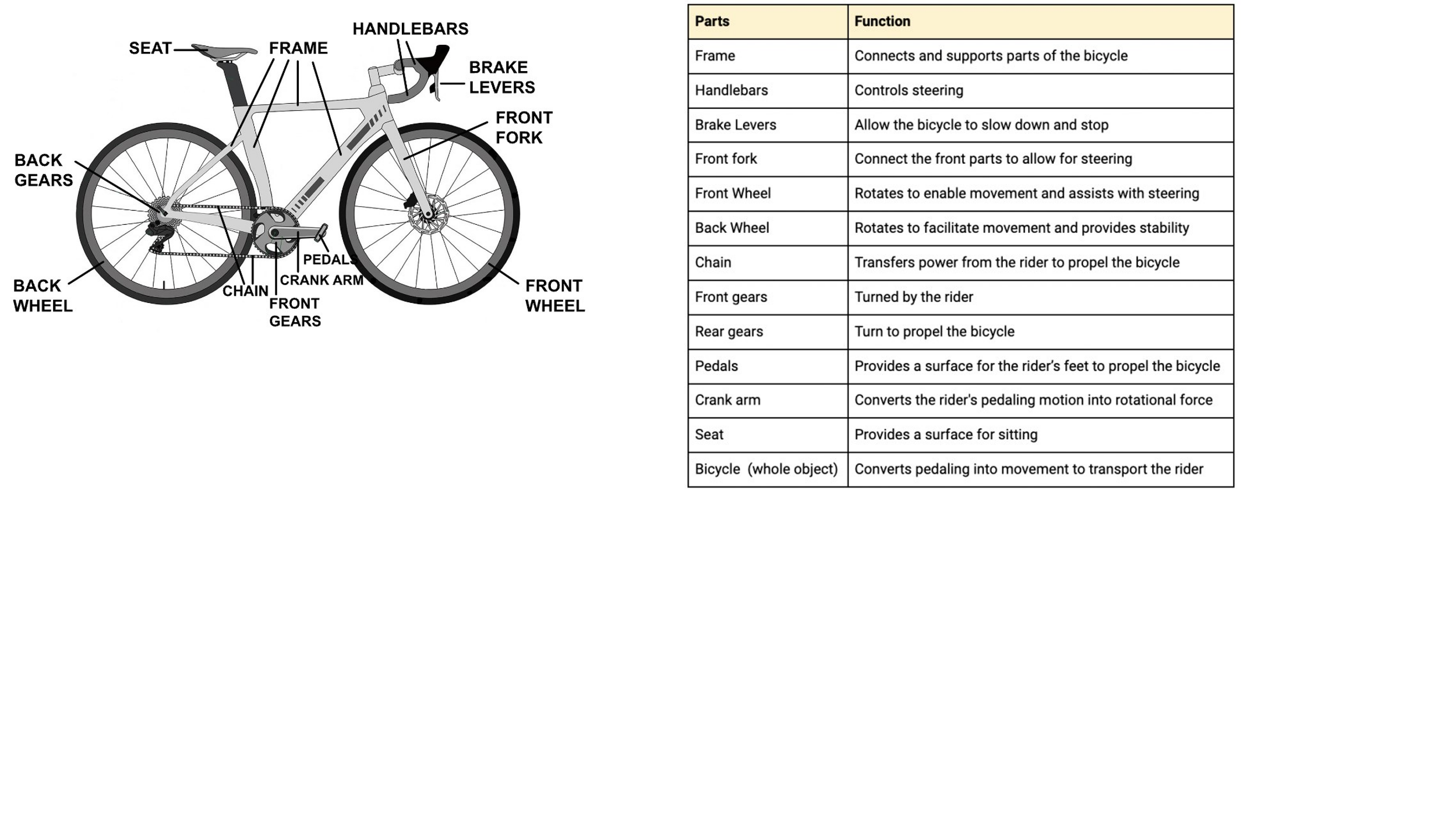
The diagram, part/function table, and the ground truth causal model for the bicycle.

**Figure S6**


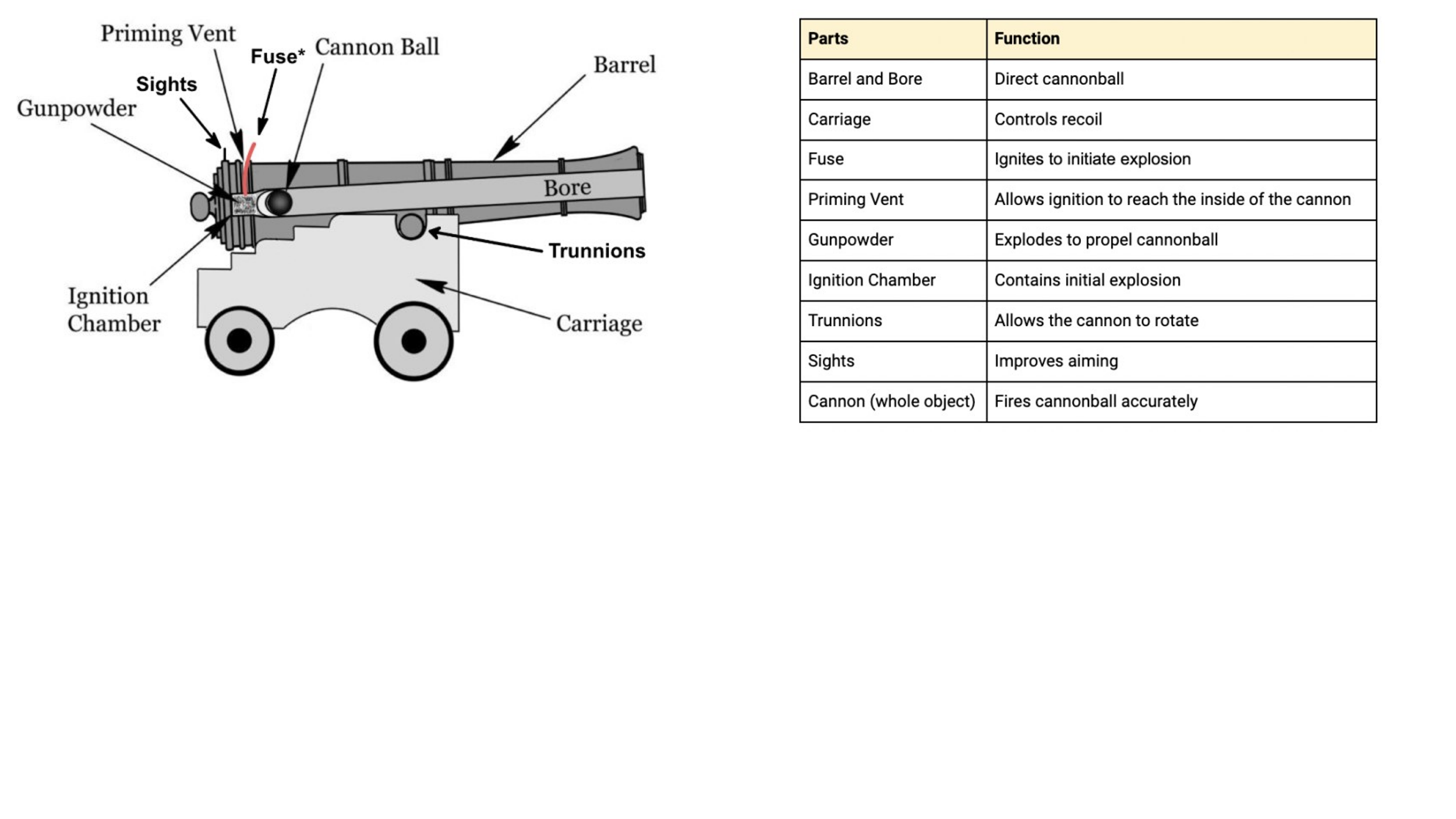

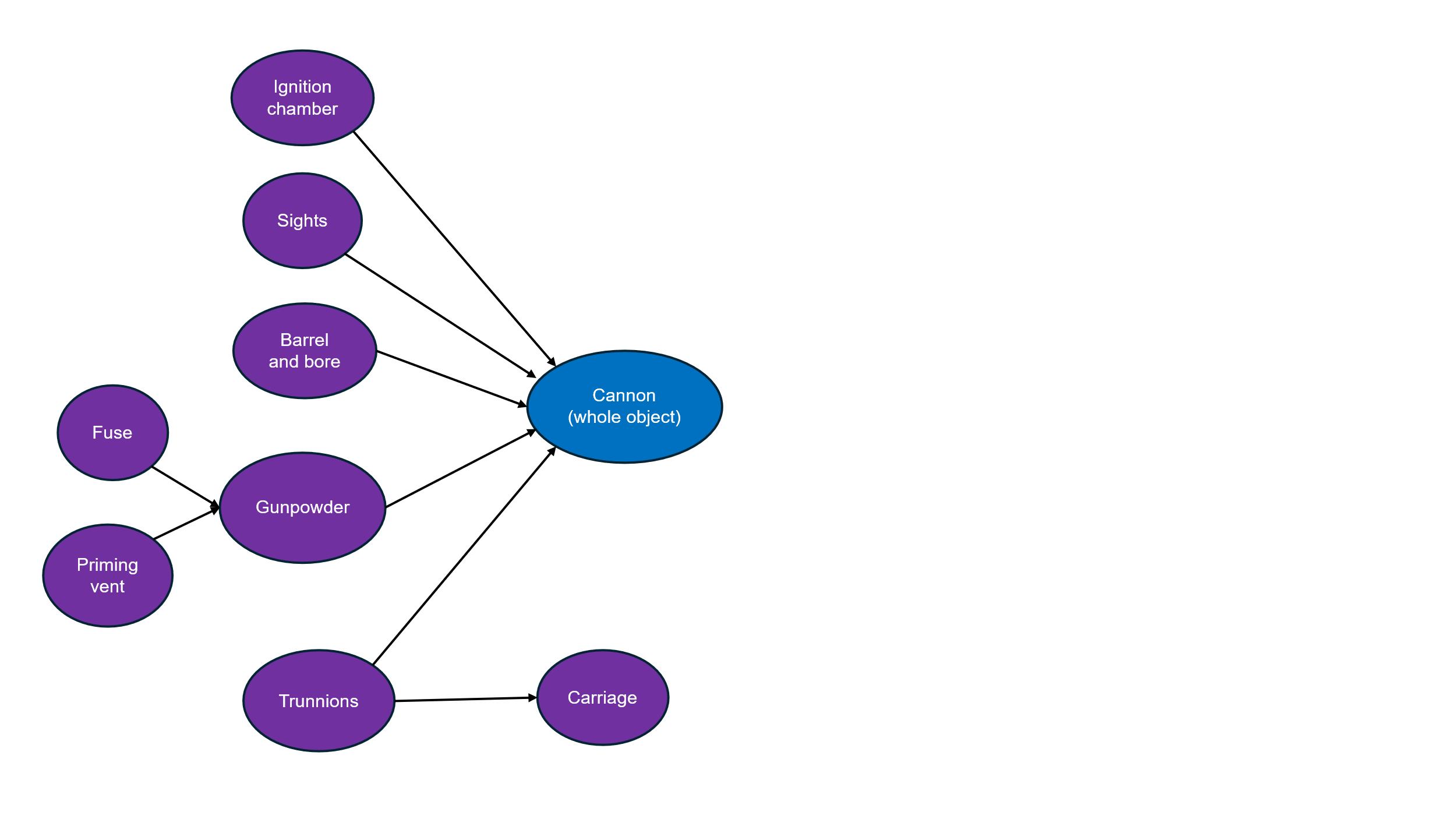
The diagram, part/function table, and the ground truth causal model for the cannon.

**Figure S7**


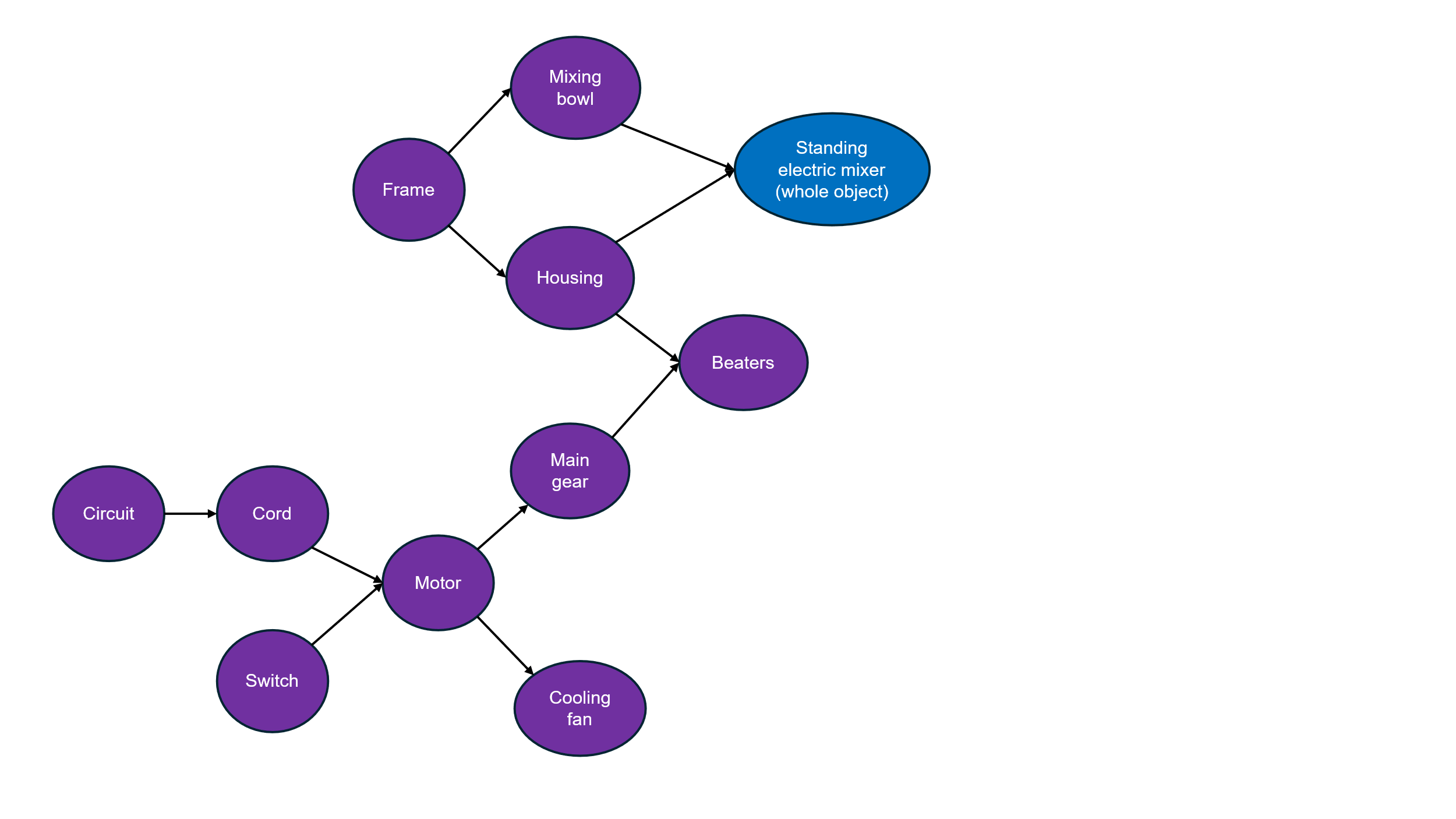

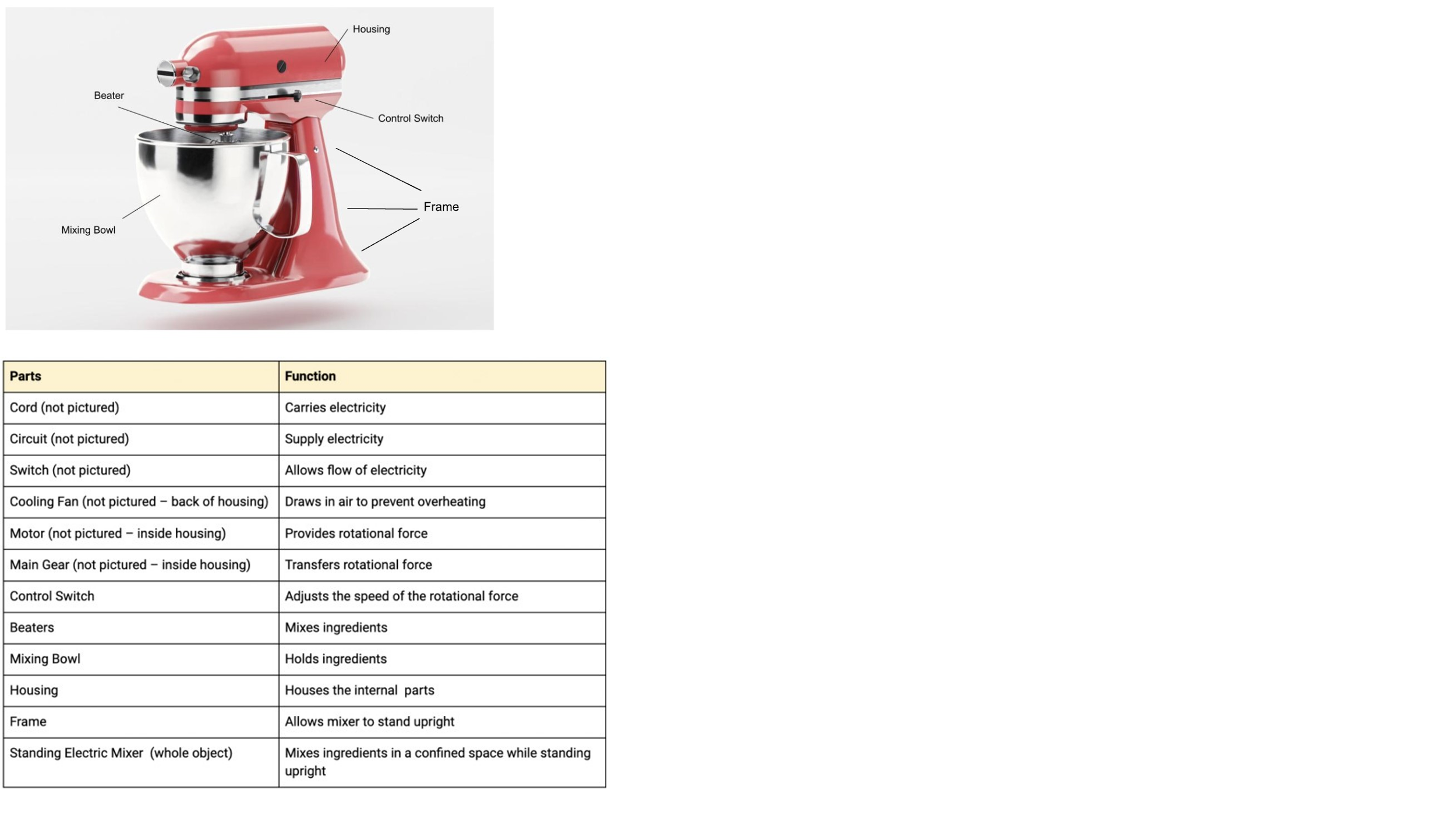
The diagram, part/function table, and the ground truth causal model for the standing electric mixer.

*Note.* Although participants saw the “control switch”, we omitted this part from their causal models as we realized it could have been confused with the “switch”.

**Figure S8**


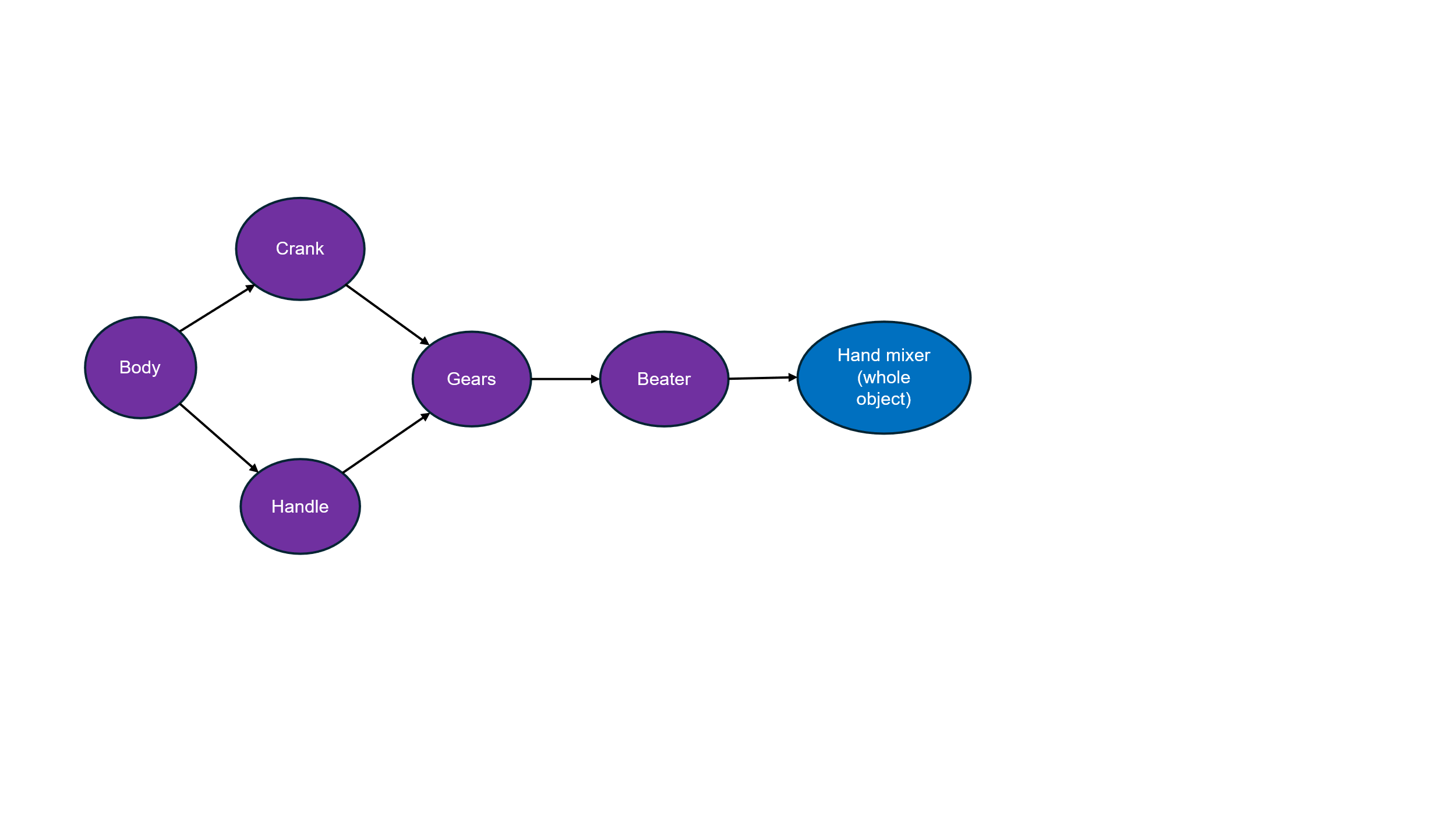

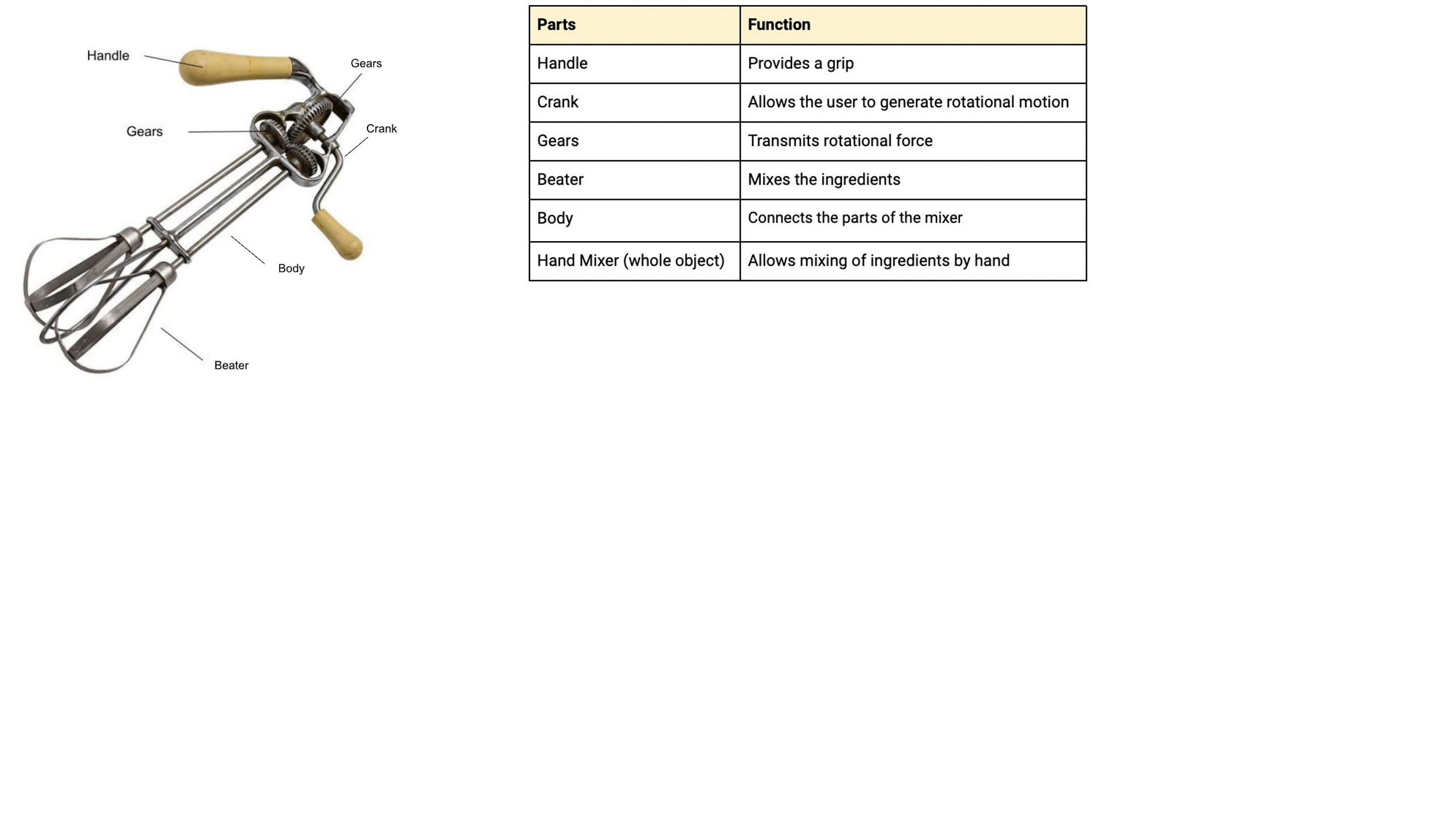
The diagram, part/function table, and the ground truth causal model for the hand mixer.

**Figure S9**


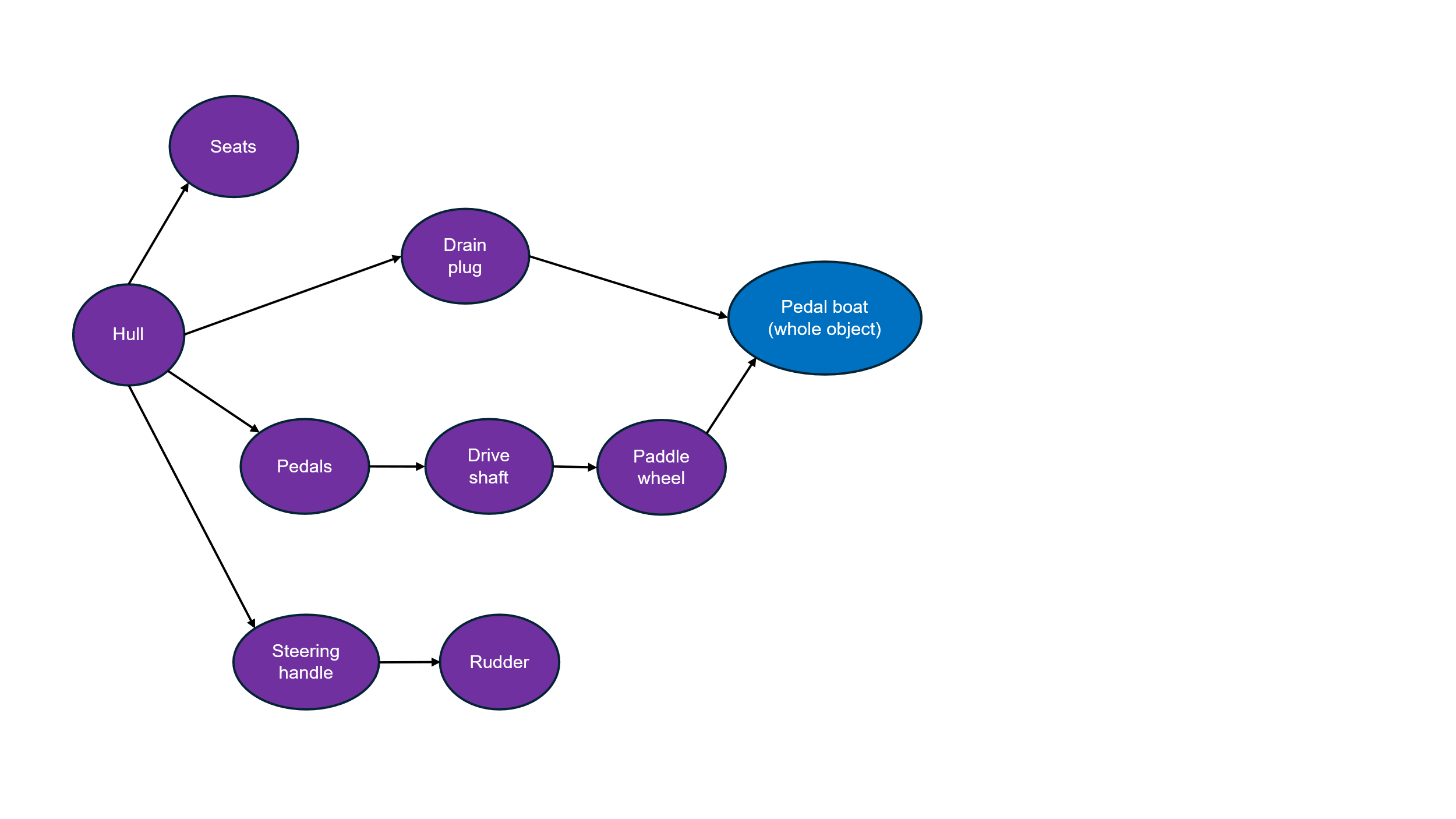

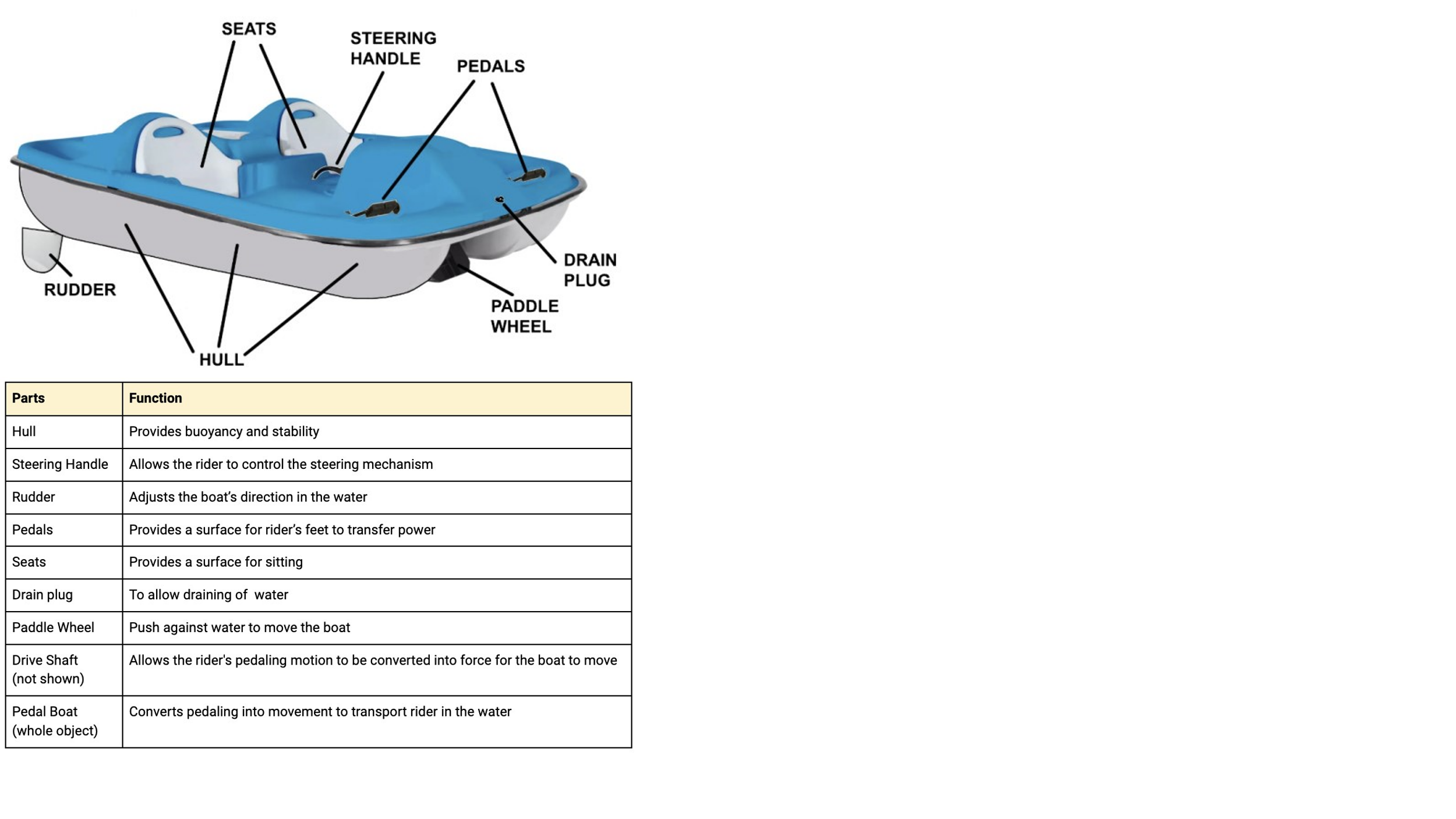
The diagram, part/function table, and the ground truth causal model for the pedal boat.

**Figure S10**


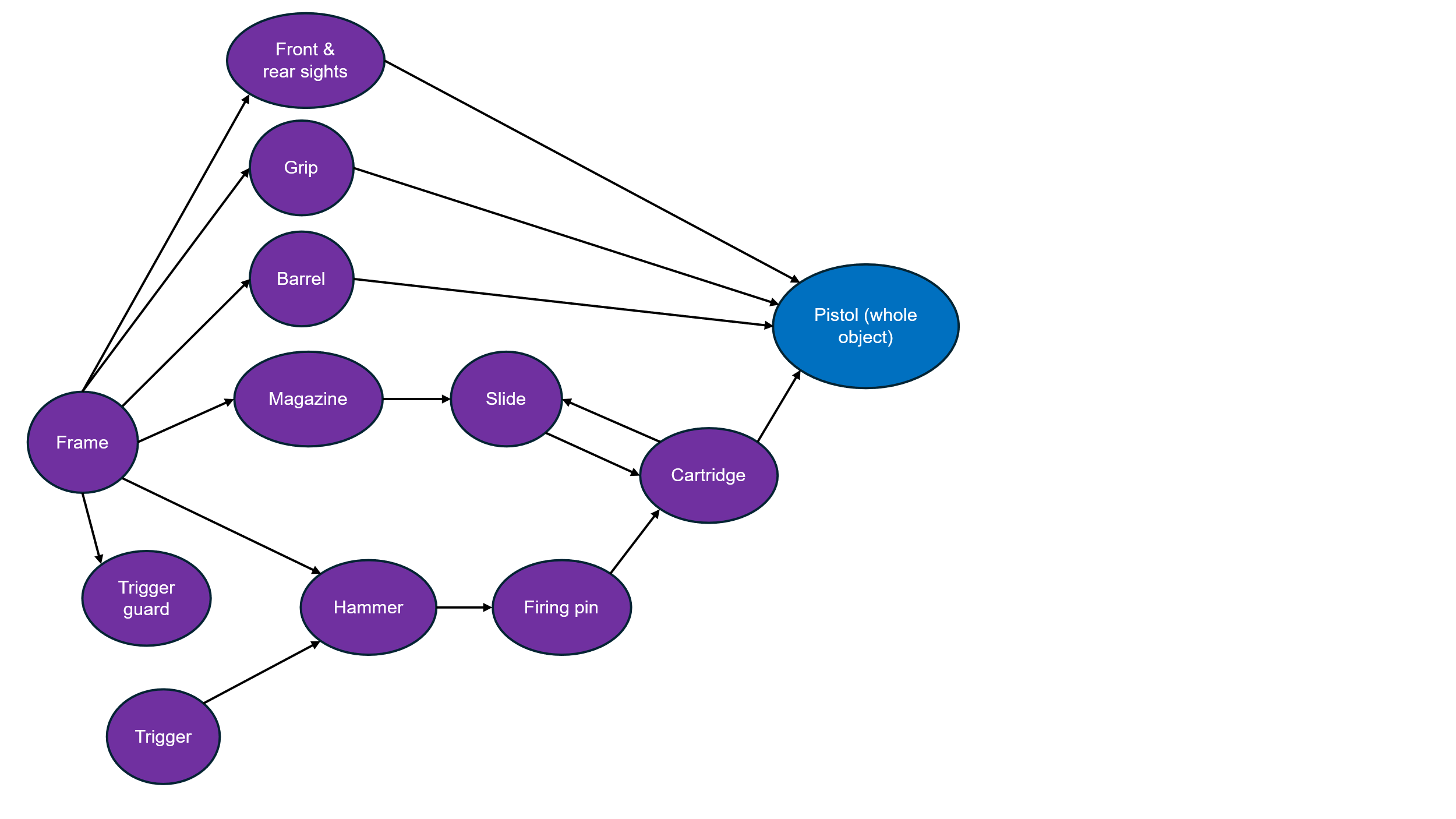

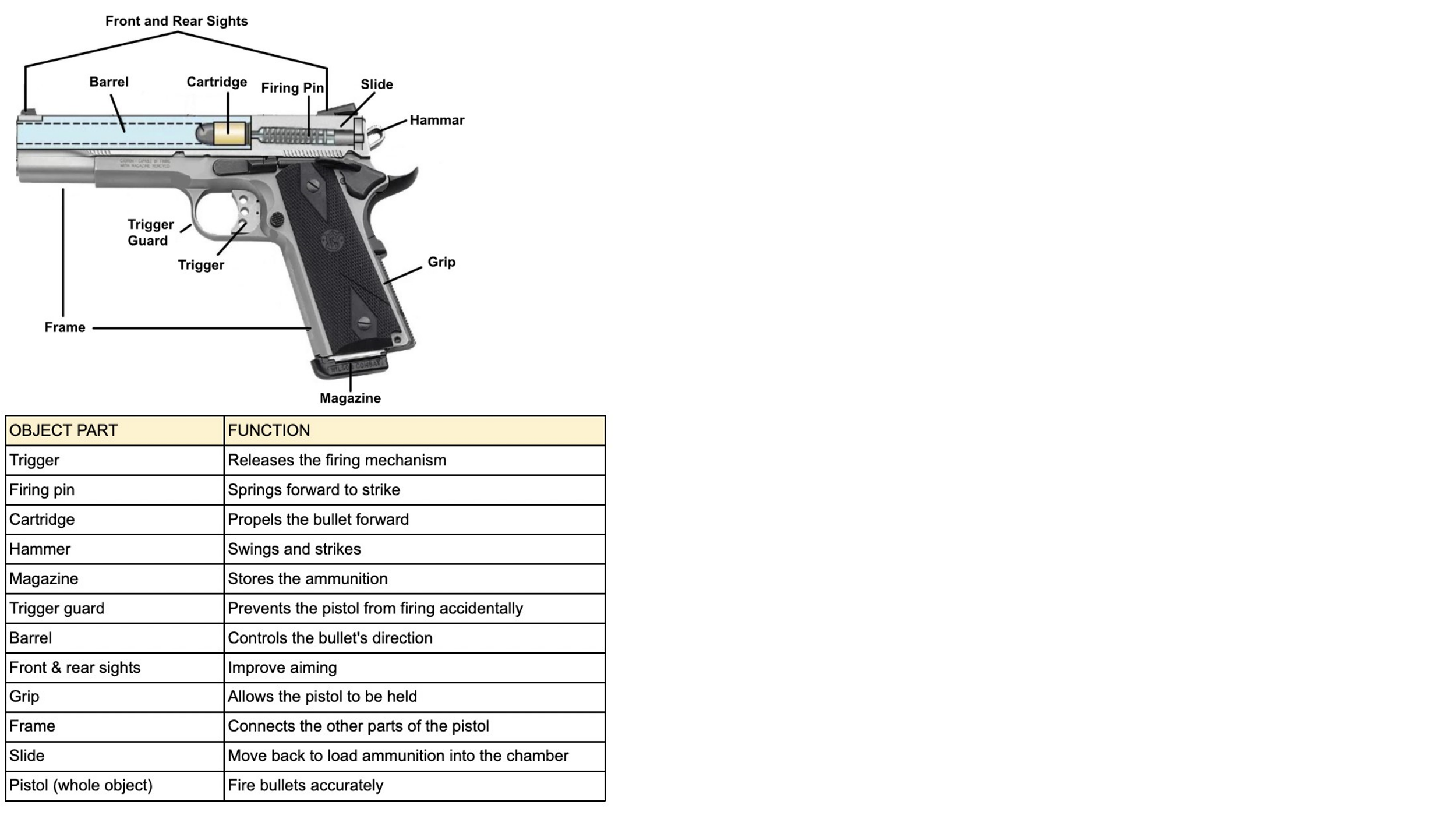
The diagram, part/function table, and the ground truth causal model for the pistol.

**Figure S11**


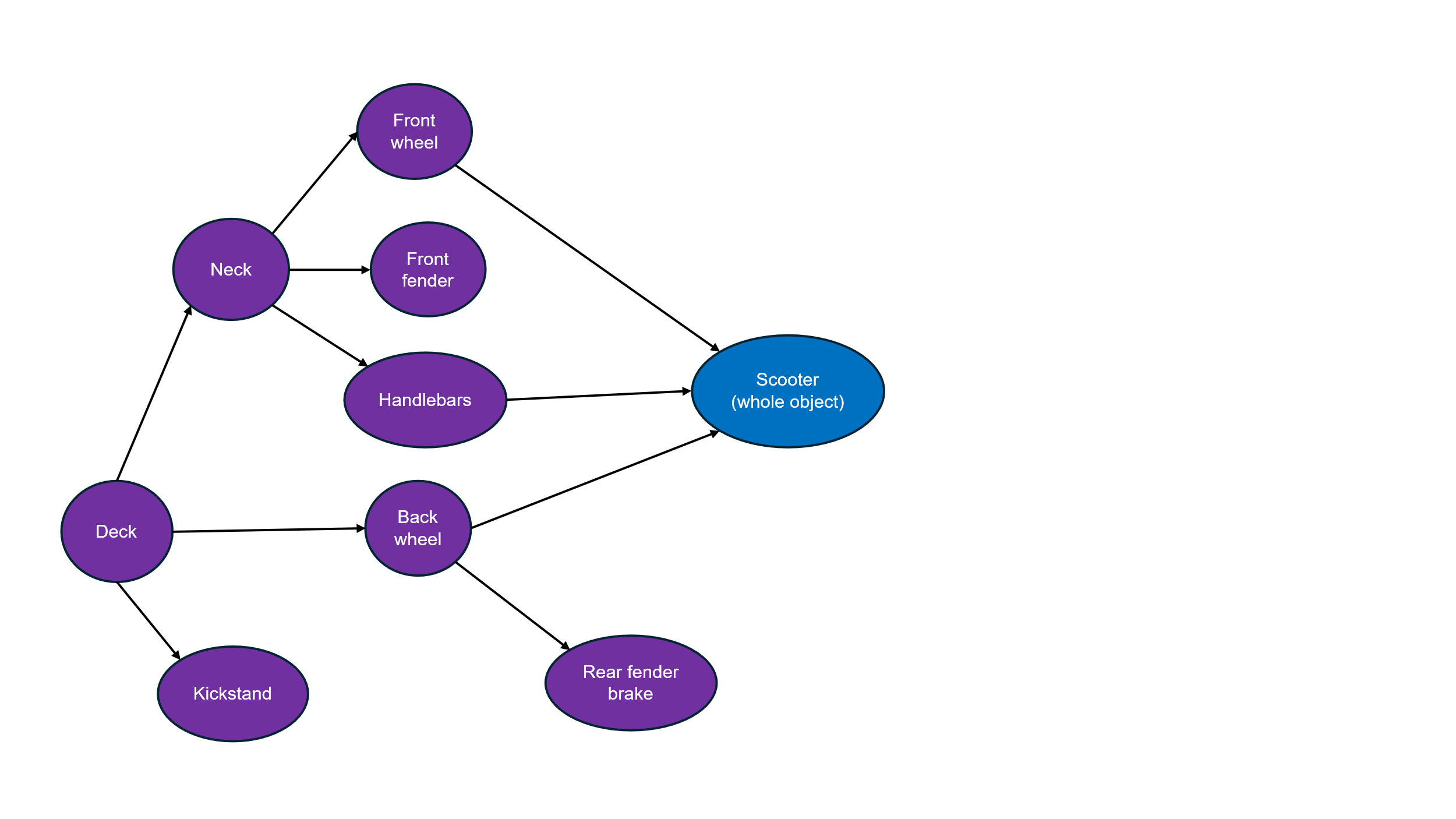

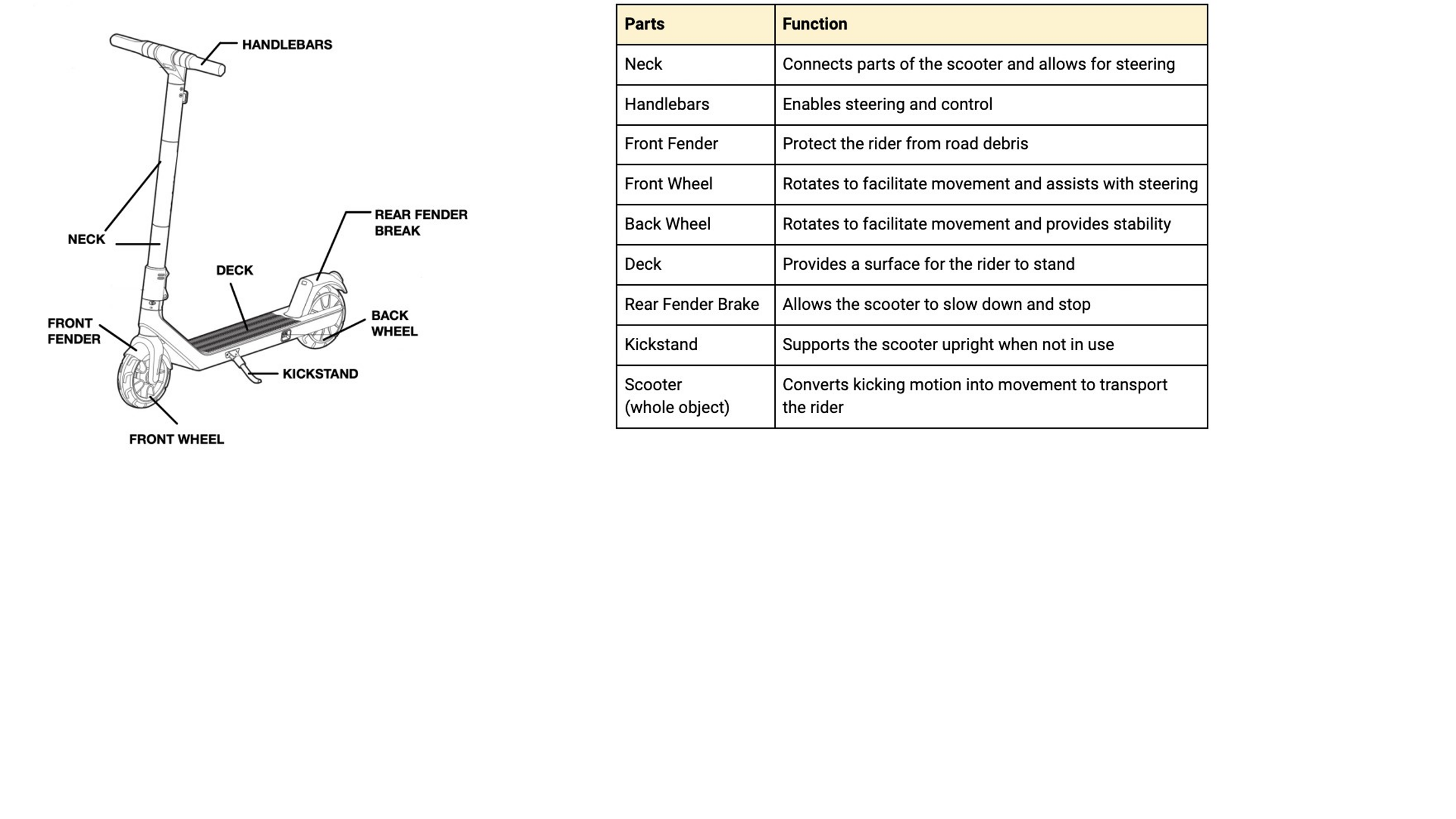
The diagram, part/function table, and the ground truth causal model for the scooter.

**Figure S12**


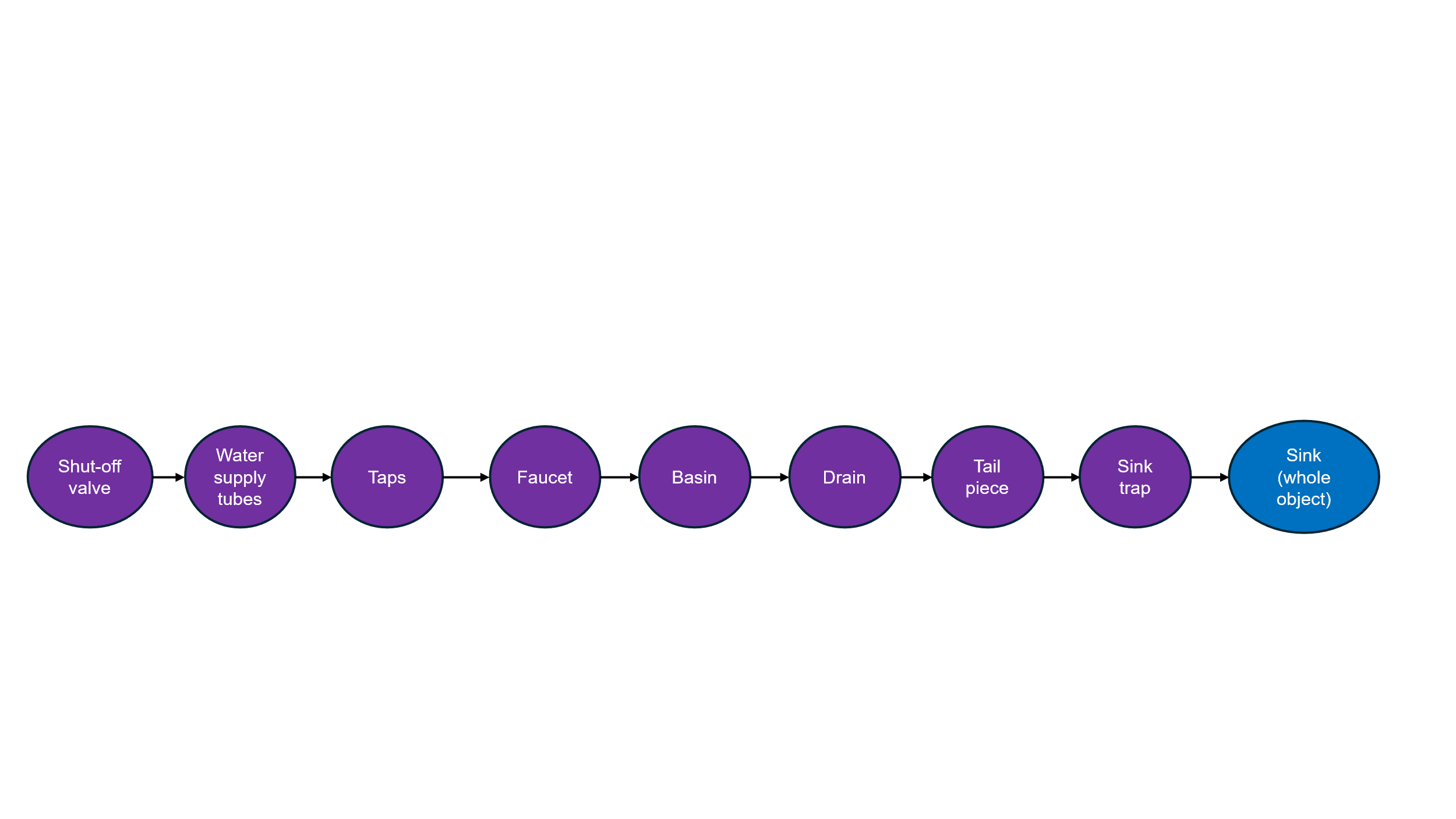

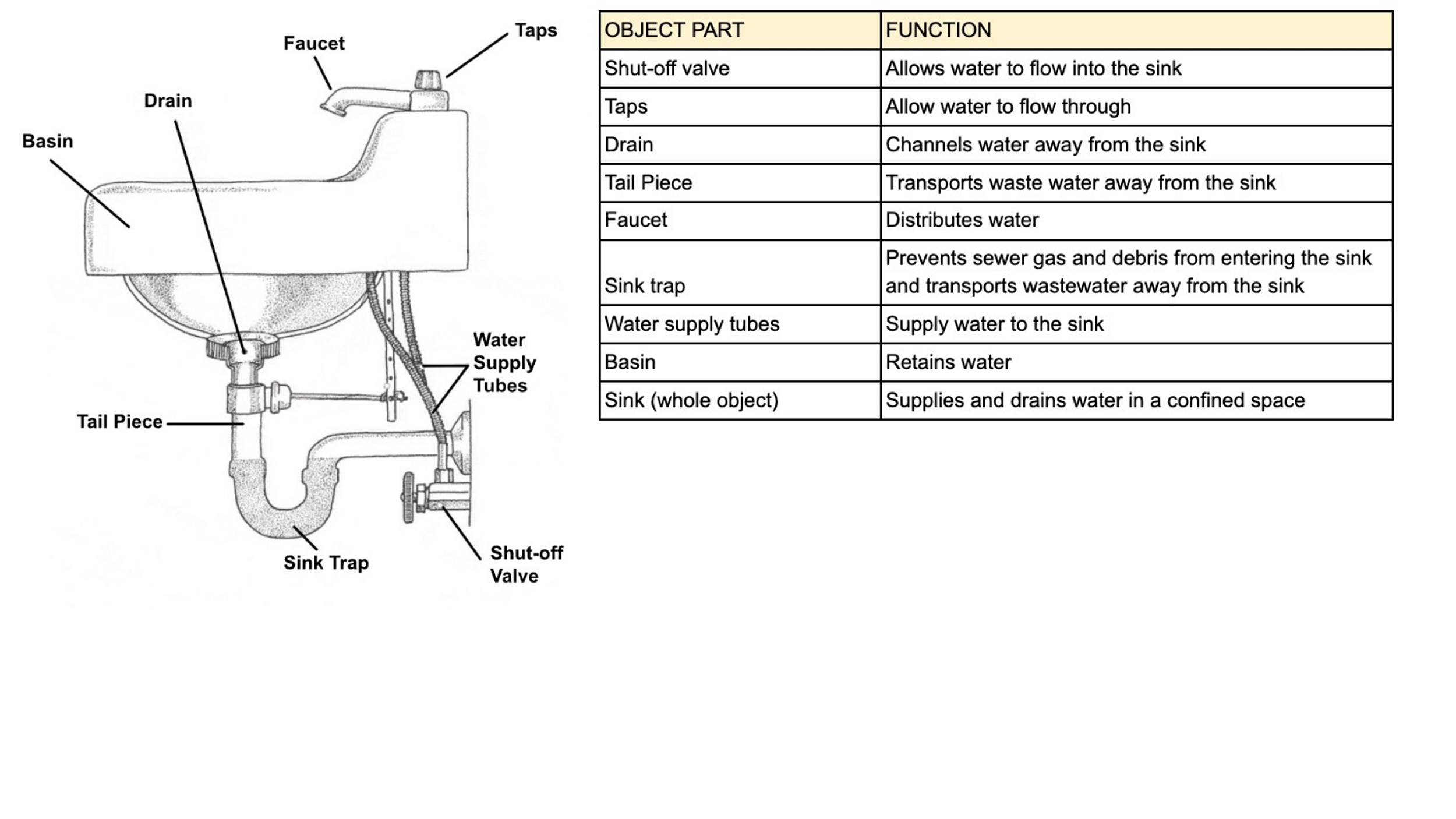
The diagram, part/function table, and the ground truth causal model for the sink.

**Figure S13**


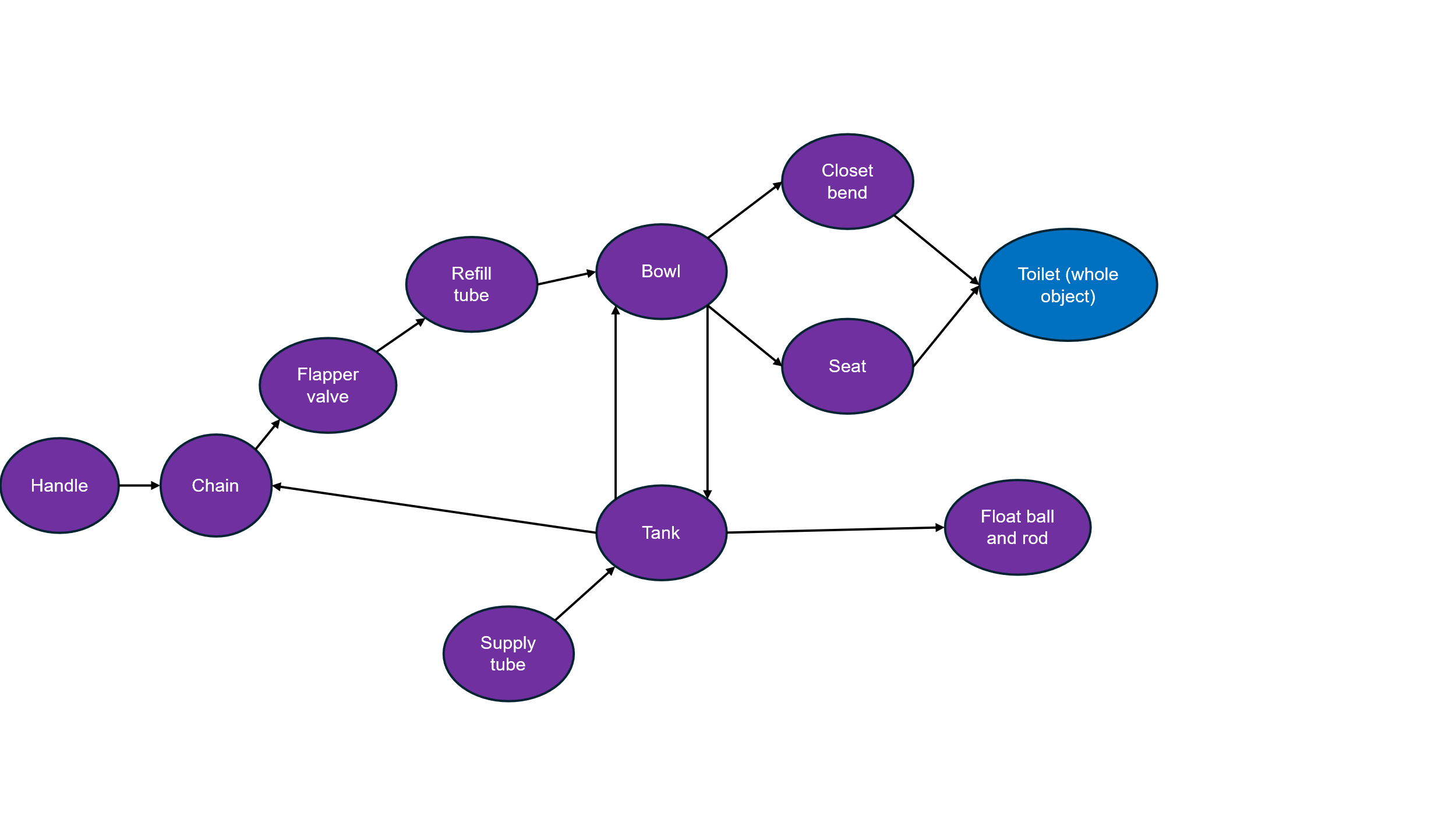

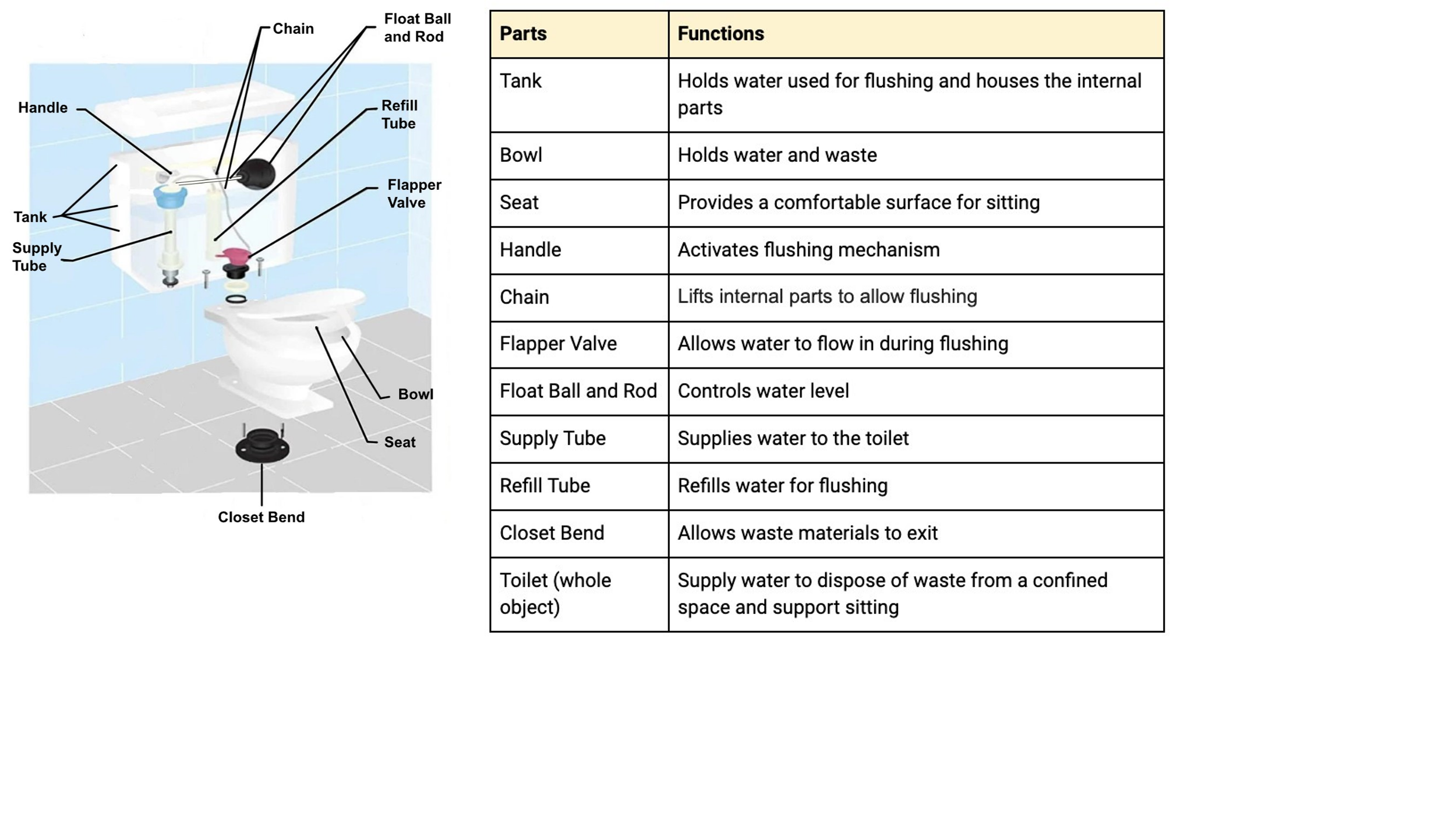
The diagram, part/function table, and the ground truth causal model for the toilet.

**Figure S14**


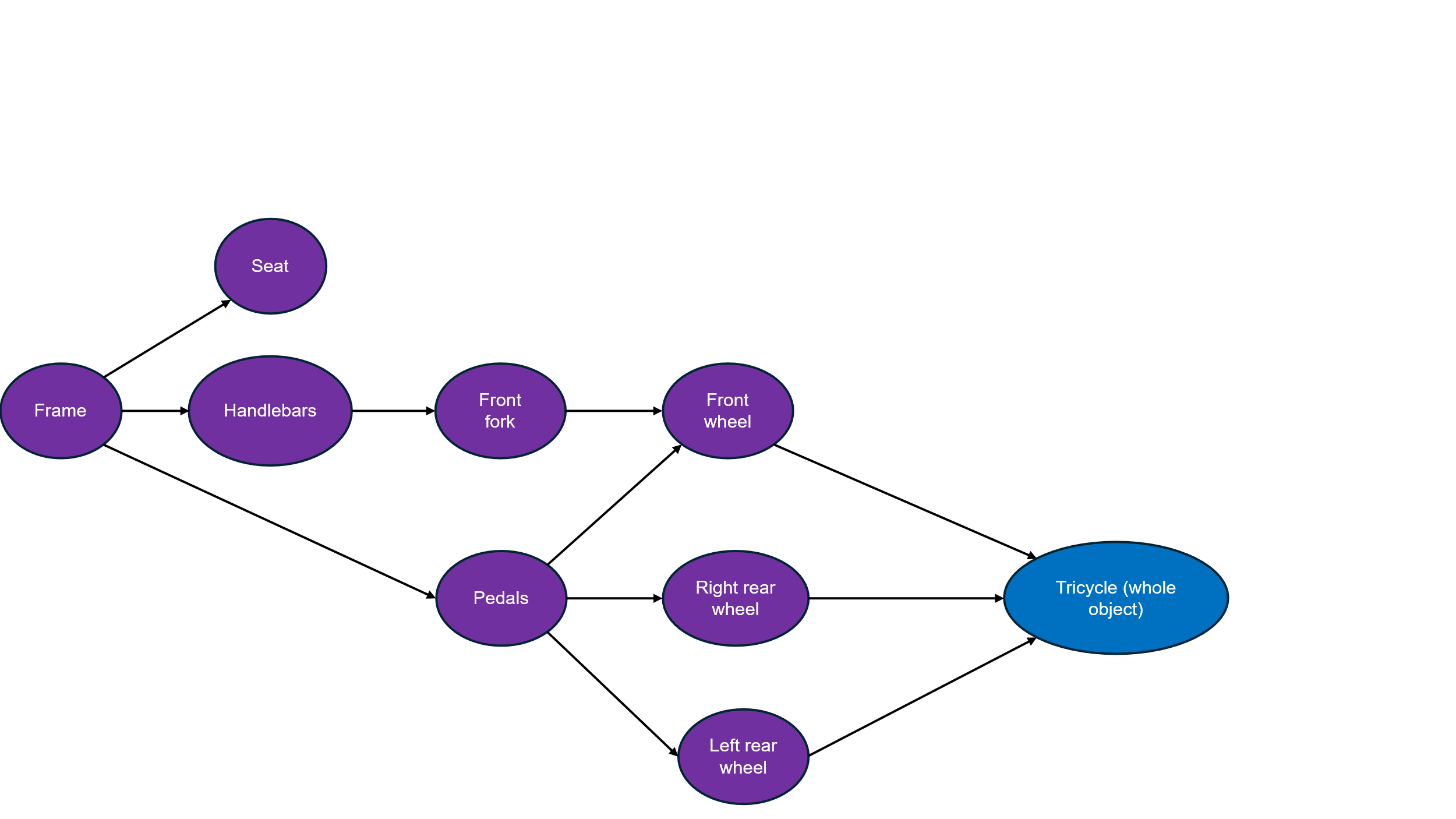

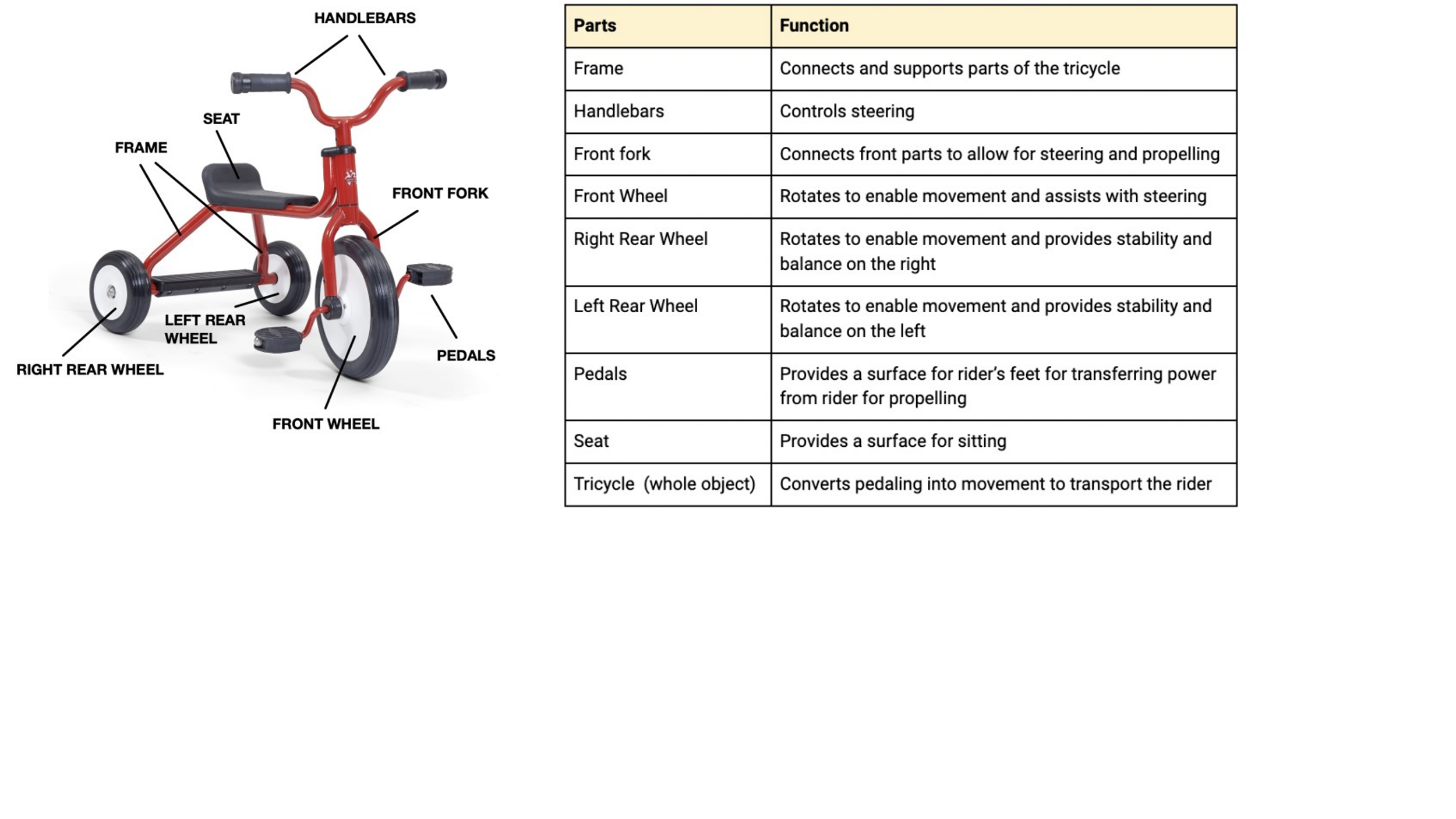
The diagram, part/function table, and the ground truth causal model for the tricycle.
